# Shifts among Eukaryota, Bacteria, and Archaea define the vertical organization of a lake sediment

**DOI:** 10.1186/s40168-017-0255-9

**Published:** 2017-04-08

**Authors:** Christian Wurzbacher, Andrea Fuchs, Katrin Attermeyer, Katharina Frindte, Hans-Peter Grossart, Michael Hupfer, Peter Casper, Michael T. Monaghan

**Affiliations:** 1 Leibniz-Institute of Freshwater Ecology and Inland Fisheries, Müggelseedamm 301, Berlin, 12587 Germany; 2 Berlin Center for Genomics in Biodiversity Research, Königin-Luise-Str. 6-8, Berlin, 14195 Germany; 3 Department of Biological and Environmental Sciences, University of Gothenburg, Box 100, Göteborg, Sweden; 4 Carl-von-Ossietzky University Oldenburg, Ammerländer Heerstraße 114-118, Oldenburg, 26129 Germany; 5 Leibniz-Institute of Freshwater Ecology and Inland Fisheries, Alte Fischerhütte 2, Stechlin, 16775 Germany; 6 Department of Ecology and Genetics, Uppsala University, Norbyvägen 18d, Uppsala, 75236 Sweden; 7 Institute of Crop Science and Resource Conservation – Molecular Biology of the Rhizosphere, Nussallee 13, Bonn, 53115 Germany; 8 Institute for Biochemistry and Biology, Potsdam University, Maulbeerallee 2, Potsdam, 14469 Germany

**Keywords:** Archaea, Eukaryota, Bacteria, Community, Freshwater, Lake, DNA metabarcoding, Beta-diversity, Sediment, Turnover

## Abstract

**Background:**

Lake sediments harbor diverse microbial communities that cycle carbon and nutrients while being constantly colonized and potentially buried by organic matter sinking from the water column. The interaction of activity and burial remained largely unexplored in aquatic sediments. We aimed to relate taxonomic composition to sediment biogeochemical parameters, test whether community turnover with depth resulted from taxonomic replacement or from richness effects, and to provide a basic model for the vertical community structure in sediments.

**Methods:**

We analyzed four replicate sediment cores taken from 30-m depth in oligo-mesotrophic Lake Stechlin in northern Germany. Each 30-cm core spanned *ca*. 170 years of sediment accumulation according to ^137^Cs dating and was sectioned into layers 1–4 cm thick. We examined a full suite of biogeochemical parameters and used DNA metabarcoding to examine community composition of microbial Archaea, Bacteria, and Eukaryota.

**Results:**

Community *β*-diversity indicated nearly complete turnover within the uppermost 30 cm. We observed a pronounced shift from Eukaryota- and Bacteria-dominated upper layers (<5 cm) to Bacteria-dominated intermediate layers (5–14 cm) and to deep layers (>14 cm) dominated by enigmatic Archaea that typically occur in deep-sea sediments. Taxonomic replacement was the prevalent mechanism in structuring the community composition and was linked to parameters indicative of microbial activity (e.g., CO_2_ and CH_4_ concentration, bacterial protein production). Richness loss played a lesser role but was linked to conservative parameters (e.g., C, N, P) indicative of past conditions.

**Conclusions:**

By including all three domains, we were able to directly link the exponential decay of eukaryotes with the active sediment microbial community. The dominance of Archaea in deeper layers confirms earlier findings from marine systems and establishes freshwater sediments as a potential low-energy environment, similar to deep sea sediments. We propose a general model of sediment structure and function based on microbial characteristics and burial processes. An upper “replacement horizon” is dominated by rapid taxonomic turnover with depth, high microbial activity, and biotic interactions. A lower “depauperate horizon” is characterized by low taxonomic richness, more stable “low-energy” conditions, and a dominance of enigmatic Archaea.

**Electronic supplementary material:**

The online version of this article (doi:10.1186/s40168-017-0255-9) contains supplementary material, which is available to authorized users.

## Background

The continuous deposition of organic and inorganic particles to sediments is an important process in all aquatic ecosystems. Approximately one third of the terrestrial organic matter (OM) that enters freshwater is sequestered in sediments [1], although the total amount of OM that reaches the sediments is much greater than the amount that is actually sequestered [2]. This is because microbial activity is responsible for the cycling of carbon, including methane emission [3]. In lake sediments, a proportion of newly settled OM is rapidly recycled and subsequently transformed into secondary compounds, resulting in a distinct uppermost sediment zone of high heterotrophic activity [4, 5]. This is thought to lead to the structuring of microbial communities along environmental gradients that are much steeper than those in marine sediments, with narrower vertical sequences of electron acceptors [6]. The nature of this gradient influences the carbon, nitrogen, and sulfur cycles [7–9] and potentially affects the microbial community structure [10].

In contrast to the wealth of studies on marine sediments (cf. the 65 studies of [11]), few studies have examined the vertical microbial community structure of freshwater sediments (e.g., [12–16]). The community of sediment microbes was thought to be dominated by Bacteria, together with a smaller fraction of methanogenic Archaea (reviews in [6, 17, 18]). This view has been challenged by the recent discovery of an abundance of non-methanogenic Archaea in marine sediments [19, 20]. They are assumed to be adapted to low-energy environments, and at least one lineage seems to be specialized in inter alia amino acid turnover [20]. This discovery has led to a revised perception of microbial communities in marine sediments, where Archaea appear to be as abundant as Bacteria and increase in relative abundance with sediment depth [11]. Data on sediment Archaea in freshwater are scarce, and the causes of the significant variation observed among studies remain largely unknown (e.g., [14, 21–23]).

Prokaryotic activity, biomass, and cell numbers decrease with depth in many freshwater and marine sediments (e.g., [24, 25]), although other studies report relatively constant proportions of active cells with depth and find no accumulation of dead cells in deeper sediments [5, 26]. Despite the continuous presence of vegetative cells and resting stages, recent studies of marine systems indicate that the majority of microbial cells in energy-deprived horizons consist of microbial necromass [27, 28] and the proportion of living organisms decreases with the increasing age of the sediment [29]. The vertical, progressive transformation of OM and depletion of electron acceptors may eventually lead to an extremely low-energy environment in deeper sediment layers with very low growth rates similar to sub-seafloor sediments [30].

A basic mechanism thought to explain the vertical distribution of microbes is simply the one-way input of new organisms attached to OM that sinks from the water column. We hypothesize that this process would result in two simplified, competing structural models, wherein the microbial community (1) consists exclusively of sinking colonizers, with the result being a fully nested community structure in which the community gradually changes from a complex and rich community at the surface to an increasingly depauperate community with increasing sediment depth, dominated by progressive cell death, or (2) is structured by niche specialists at various layers that are well adapted to the specific environmental conditions including redox gradients, OM, and nutrient (C, N, P) concentrations. These two models are suitably analogous to the recently developed *β*-partitioning of the total *β*-diversity of a community, in which the taxonomic turnover is mathematically separated into richness and nestedness components (see [31] for a theoretical framework and [32] for applications).

The decomposition rate of settled or buried pelagic dead and living organisms is thought to depend primarily on the activity of the indigenous microbial community rather than on chemical processes (e.g., depurination; [33]). The decrease in DNA with depth that has been reported for freshwater sediments (e.g., [34]) is likely to result of nucleic acid degradation of dead organisms, particularly eukaryotes, whose biomass also decreases with depth. As a result, the decomposition of buried organisms should be a function of the active community which is itself buried over time. Vice versa, temporal patterns of sedimentation will also influence the active microbial community, for example by shifting the redox gradient. Historically changing lake conditions are recorded in lake sediments as DNA and as chemical parameters (e.g., [34]). An important question that remains is how decomposition processes within the sediment redox gradient are related to the burial of OM, eukaryotes, and prokaryotes [35, 36].

We examined the biogeochemical properties and microbial community composition (Eukaryota, Bacteria, and Archaea [37]) of sediments in the oligo-mesotrophic hardwater Lake Stechlin in northeast Germany. Our aims were to evaluate (1) whether microbial communities were nested or structured (to test the competing models, above), (2) how sediment parameters reflecting “present” and “past” conditions influence the overall community structure (Table 1), and (3) whether recently reported vertical patterns of marine Archaea [11] can predict those observed in freshwater sediments. We took four replicate 30-cm sediment cores from ca. 30-m water depth. ^137^Cs dating indicated the cores include sediments deposited over the past ca. 170 years.

**Table 1:** Definition of “present” and “past” sediment parameters

We define *present* parameters as the principal components of all context
data derived from (a) pore water analysis, which indicates that chemical
gradients are caused by the consumption and production of ongoing
biological processes (e.g., sulfate and methane), and from (b) directly
measured parameters of microbial activities (e.g., bacterial protein
production). The present parameters are therefore an expression of
recent microbial processes.
*Past* parameters are the principal components of conservative
parameters, which once introduced into the sediments will not change
significantly and are therefore an expression of the lake’s history (e.g.,
heavy metals). Here, we also categorize the total amount of elemental
carbon, nitrogen, hydrogen, and sulfur as mainly conservative parameters.
The past parameters are therefore an expression of historical changes.

## Results

**Fig. 1:**
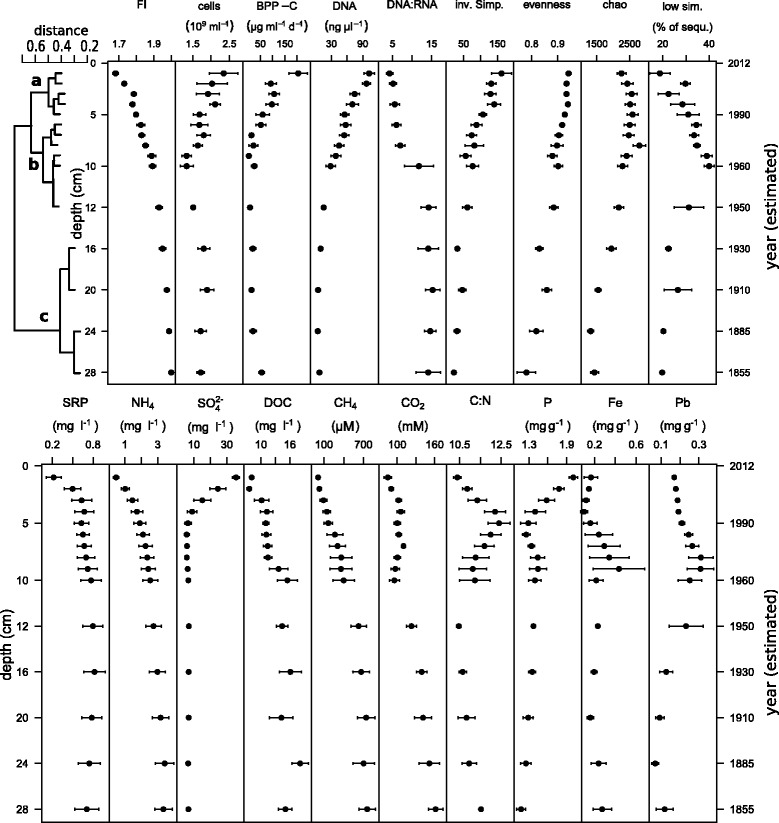
Depth profiles of microbial community clustering and key biological and chemical characteristics of the sediment cores taken from 30-m depth in Lake Stechlin. Microbial communities were clustered by similarity (average clustering) into three groups (a, b, c) corresponding to different depth horizons (*upper-left panel*). Dates (*y*-axis) were calculated using ^137^Cs measurements. Values are means (±1 SE) from four replicate cores. Parameters and units: FI = fluorescence index; cells [ 10^6^ ml^−1^]; BPP-C = bacterial protein production in carbon [ μg C ml^−1^d^−1^]; DNA extract [ng *μ* *l* ^−1^]; the shared chao index (R vegan package, [106]); low sim. = proportion of sequences with no close relative [% of sequences]; SRP [ mg l^−1^]; NH_4_ [ mg l^−1^]; SO$_{4}^{2-}$ [ mg l^−1^]; DOC [ mg l^−1^]; CH_4_ [ μmol l^−1^]; CO_2_ [ mmol l^−1^]; P [ mg g^−1^ dry weight]; Fe [ mg g^−1^ dry weight]; Pb [ mg g^−1^ dry weight]. Additional information for all measured variables per individual core are provided in the Additional files 2 and 12

Sediments in the cores were black in color, with no visible lamination. Water content was 93–97%. No macrozoobenthic organisms were visible, although DNA metabarcoding (see below) detected the presence of nematodes in addition to microbes (Additional file 1). There was an exponential increase of dissolved refractory carbon with sediment depth (fluorescence index (FI) = 1.69–2.01; Fig. 1) across all four cores, indicating the enrichment of fulvic acids [38]. Prokaryotic cell numbers were on average 1.8 ± 0.5×10^9^ ml^−1^ wet sediment and were highest in the upper sediment layers. Bacterial biomass production as carbon (BPP-C) (range 0–282 μg C ml^−1^d^−1^) decreased rapidly with depth, approaching zero below 10 cm. Total DNA concentration (range <0.3–17.6 μg ml^−1^ sediment) was negatively correlated with FI (*r*=−0.886) and followed an exponential decay function. DNA half-life was inferred to be *t*_1/2_=22 *a* (corresponding to 5.4 cm; *f*(DNA) = 13.9 × *e* ^−0.128*x*^,*r* ^2^=0.81). RNA content was lower than DNA content in all layers, with DNA:RNA ratios ranging from 2.3 at the surface to 20.8 at 20-cm depth (Fig. 1). The sediment exhibited a typical electron acceptor sequence (Fig. 1) with a mean oxygen penetration depth of 4.6 mm (SD 1.4). Nitrate and nitrite were immediately depleted at the sediment surface, sulfate approached a constant minimum concentration after 5 cm, soluble reactive phosphorous (SRP) and ammonium (NH$_{4}^{+}$) increased with sediment depth, N_2_O gas was not detected, CH_4_ increased linearly with depth, and CO_2_ exhibited minima at the surface and at a depth of 10 cm (Fig. 1). More detailed profiles of all measured parameters can be found in the supplemental material (Additional file 2).

Total taxon richness across the 60 samples was estimated (Chao) to be 8545 (SE = 173) operational taxonomic units (OTUs). The proportion of sequences with no close relatives in the SILVA reference database (<93*%* sequence similarity using BLAST) was highest at a depth of 9-10 cm (40±4*%*). An overview of eukaryotic, bacterial, and archaeal OTUs recovered in each sample layer is provided in Additional file 1.

**Fig. 2:**
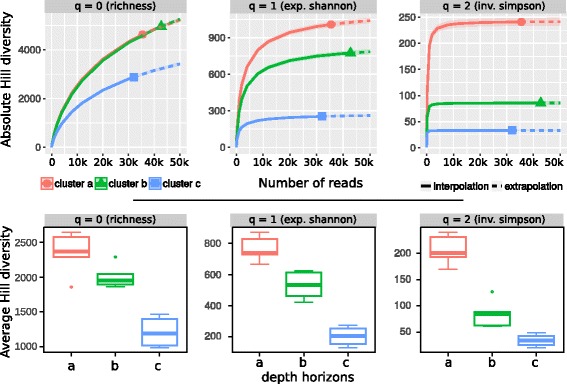
Hill diversities of the three depth horizon clusters. Absolute (*upper panel*) and average (*lower panel*) Hill diversity for the three Hill numbers *q* resembling richness (0), exponential Shannon Index (1), and Inverse Simpson index (2)[39]. The absolute data is based on all sequences obtained for each horizon; the average diversity is based on all sequences from each sampling depth, grouped as horizons, normalized to a sampling coverage of 0.9. The average diversity was significant different between depth horizons for all Hill numbers, ANOVA: *F*(2,12)=27.9,59.8,67.5 for *q*=0,1,2; respectively; *p*<0.001

When pooling all replicates for analysis of the community matrix with a cluster analysis, the microbial communities were grouped into three major clusters corresponding to depths of 0–5, 5–14, and 14–30 cm, with a pronounced separation at 14 cm (Fig. 1). The sediment communities in layers between 14–30-cm depth were more similar to one another (>65*%* of community structure) than layers in the upper two clusters (<50*%*). All *α*-diversity indices (inverse Simpson, evenness, and estimated Chao index) decreased with depth (Fig. 1). This pattern also occurred in the rarefaction analysis of the Hill indices [39] and confirmed a significant separation of the three depth clusters by major taxonomic changes (Fig. 2). Community turnover (distance) increased with depth, following a distance decay curve and approaching a distance of 1 (i.e., no shared taxa) for the comparison of the lowest layer (30-cm depth) with the surface (Table 2). Upon partitioning the *β*-diversity among sample layers into taxonomic richness and replacement effects [32], taxonomic replacement was consistently high and was significant for multiple sample layers above 12 cm (Table 2). In contrast, the effect of richness increased with depth (*R* ^2^=0.96,*F*=230.8,*dF*=11, see Additional file 3) and it was significantly elevated in the deepest layer (26–30-cm depth) (Table 2).

**Table 2:** Microbial community turnover and richness for each sampling layer

Depth [cm]	Distance	Repl.	Richness	LCrepl	LCrich	OTUs	Coverage
0–1	NA	NA	NA	0.083***	0.039	1787	0.825
1–2	0.683	0.600	0.083	0.086***	0.001	1566	0.867
2–3	0.668	0.662	0.006	0.068	0.050	1724	0.828
3–4	0.688	0.659	0.029	0.057	0.079	1870	0.818
4–5	0.735	0.733	0.003	0.064	0.052	1759	0.833
5–6	0.739	0.709	0.030	0.070*	0.024	1625	0.859
6–7	0.761	0.722	0.040	0.069	0.019	1611	0.860
7–8	0.780	0.751	0.029	0.066	0.030	1709	0.841
8–9	0.822	0.749	0.072	0.071**	0.010	1567	0.861
9–10	0.824	0.753	0.071	0.071**	0.010	1557	0.863
10–14	0.860	0.782	0.078	0.074***	0.011	1518	0.853
14–18	0.902	0.720	0.183	0.063	0.075	1268	0.880
18–22	0.920	0.715	0.206	0.061	0.113	1242	0.891
22–26	0.930	0.689	0.241	0.056	0.183	1087	0.905
26–30	0.934	0.649	0.285	0.041	0.304*	1019	0.903

*Distance* total Jaccard-based distance measures between surface (0–1 cm) and each lower layer, *Repl.* replacement component of the Jaccard distance, *Richness* richness component of the Jaccard distance, *LCRepl* local contribution of the replacement component, *LCRich* local contribution of the richness component, *OTUs* number of observed OTUs, *Cov.* sample coverage. Asterisks indicate significantly increased LC values with *p*<0.05*, 0.01**, and 0.001***, respectively

**Fig. 3:**
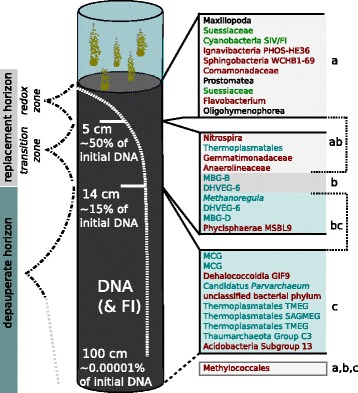
Overview of the sediment structure in Lake Stechlin. The cluster analysis separates three depth horizons: the *redox-stratified zone* (0–5 cm), which includes a thin layer of oxygen. A few fauna species exist in this zone, i.e., Nematoda, Gastrotricha, and microeukaryotes (e.g., Ciliophora), in addition to large numbers of highly active Bacteria. Below 5 cm, where 50% of the DNA is already decomposed, the system enters the *transition zone*. This zone is situated below the sulfate-methane transition. Below 14 cm, we find the *depauperate horizon*, which extents in the deeper sediment, in which Archaea dominate the community. In an extrapolation of the richness component of the community structure, the loss of richness would completely dominate (100%) the microbial community at 1-m depth (approx. 500 *a*). Following the decay curve of the DNA, 99.99999% of the DNA would be transformed at that depth. On the right side, the ten most structuring OTUs (from Additional file 4) are listed, which were significantly elevated in the corresponding horizon (only results with *p*<0.01 in the Tukey HSD post hoc test were included). The brackets ab and bc mark those OTUs that were elevated in the upper two or lower two zones, respectively. Only two OTUs were elevated in the transition zone. The gray box marks the single taxon that was significantly different in all three horizons. Taxon names are color coded according to their classification or phototrophy if applicable: phototrophic organism (*green*), Eukaryota (*black*), Bacteria (*red*), and Archaea (*blue*)

The OTUs that were the most influential in structuring the microbial community across our 15 sediment layers were identified by calculating species (OTU) contribution to *β*-diversity [32]. This analysis identified 96 “structuring” OTUs (see Additional file 4) whose identity reflected the interrelationship of domains. The most influential phyla were Euryarchaeota and Thaumarchaeota (Archaea) as well as Chloroflexi, Proteobacteria, and Phycisphaerae (Bacteria) (Additional file 4). Structuring OTUs included both redox-dependent groups (13% of structuring OTUs could be clearly assigned to redox processes by their classification, e.g., Nitrospiraceae, Desulfobacteraceae, and Methylococcales) and redox irrelevant groups (e.g., Eukaryota, Bacteriovoraceae). A number of the 96 structuring OTUs were significantly elevated in one or more zones (Fig. 3). Eukaryotic and bacterial lineages were characteristic for the uppermost cluster (cluster a in Fig. 1), whereas Archaea and Bacteria were elevated in the lowest zone (cluster c) (Fig. 3). For cluster b, only two structuring archaeal OTUs were identified. The residual OTUs from cluster b were significantly elevated either in the upper two clusters (mainly Bacteria) or in the lower two clusters (mainly Archaea). Only one structuring OTU (Methylococcales) was significantly different in its relative abundance in all three clusters (Fig. 3).

**Fig. 4:**
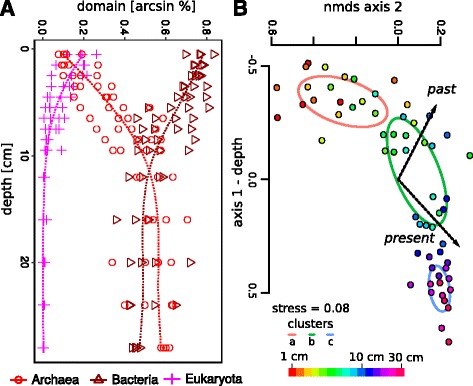
Microbial proportions and community structure. **a** Depth profiles of the microbial community (Eukaryota, Bacteria, and Archaea) presented as relative proportions to each other, which was determined by relative pyrosequencing reads per microbial fraction. **b** NMDS ordination of the vertical sediment microbial community structure. The clusters from Fig. 1 are presented as standard deviation around the group centroid. The color scale of the *dots* represents sediment depth. Community composition was highly correlated with sediment depths (on axis 1; Mantel correlation: *r*=0.735,*p*<0.001). The three distinct clusters at different depths were significantly different (adonis, *p*=0.0005) with a reduced variance in cluster c (Tukey’s honest significant differences between groups, *p*<0.01). The principal components of “past” and “present” environmental parameters were correlated with the community matrix (Mantel correlation: “present” *r*=0.527,*p*<0.001 and “past” *r*=0.459,*p*<0.001) and with the ordination (envfit: “present” *R* ^2^=0.658,*p*<0.001 and “past” *R* ^2^=0.547,*p*<0.001)

Sequence proportions of Archaea, Bacteria, and Eukaryota (A:B:E) shifted from 10:70:20 at 0 cm to 50:50:0 at 10 cm and 60:40:0 at 30-cm depth (Fig. 4 a). The eukaryotic proportions were correlated with DNA concentration (*r*=0.869) and decayed exponentially with depth. Multiple linear regression could predict DNA concentration as a function of the occurrence of Eukaryota (75.6% of the variation) and Bacteria (10.0% of the variation; model: *R* ^2^=0.856,*p*<0.001, Additional file 5). A multivariate ordination of all samples confirmed the strong vertical gradient in microbial community structure, reflected in the distance between the surface and deep sediments on axis 1 (Fig. 4 b, Mantel correlation: *r*=0.735,*p*<0.001). The three distinct clusters at different depths (above) were recovered using adonis (Fig. 4 b, *F*=12.3,*p*=0.0005). The variance in cluster c was reduced compared to the other clusters (betadispersal analysis: Tukey’s honest significant differences between groups, *p*<0.01), confirming the greater similarities seen in the previous cluster analysis. Microbial community structure was correlated with sediment parameters representative of both “present” (Mantel correlation: *r*=0.527,*p*<0.001) and “past” (*r*=0.459,*p*<0.001) conditions (see Table 1). These two parameter sets were nearly orthogonal in ordination (Fig. 4 b). Apart from the betadispersal analysis, we came to the same conclusion when we applied weighted phylogenetically based UniFrac distances instead (significant structuring along the depth gradient, significant separation of the three depth clusters, significant correlation with the “present” and “past” principal components with comparable effect sizes; see Additional file 6).

In order to examine links between the two parameter types and microbial community structure, we used fuzzy set analysis to test whether past parameters were correlated with the richness component and present parameters with the replacement component of the microbial community. We partitioned the whole dataset into replacement and richness matrices and correlated these with the present and past parameters. The richness community submatrix was strongly correlated with the past parameters (two-dimensional fuzzy set ordination with the first two axes of the PCA, *r*=0.99), and the replacement community submatrix was correlated with the present parameters (one-dimensional fuzzy set ordination with the first axis of the PCA, *r*=0.65,*p*<0.001).

## Discussion

Comprehensive studies of the microbial communities and physical-chemical characteristics of freshwater sediments are scarce, and general concepts are often transferred from marine systems without validation. This is despite the fact that salinity and sulfate concentrations are very different in the two ecosystem types [40, 41] and can have a profound influence on biological communities and biogeochemical processes. We addressed fundamental questions regarding the vertical structure, organization, and inter-relationships among microbial communities and biogeochemical parameters in freshwater sediments and establish a structural model that can potentially be applied to other aquatic sediments.

### Vertical organization of Lake Stechlin sediments

The sediment habitat is thought to be autonomous in terms of species richness and community structure [17, 18], despite the constant colonization by microbes that descend from the water column with sinking organic particles (Additional file 4). In Lake Stechlin, we observed a high species (OTU) *β*-diversity with depth in lake sediment, leading to nearly complete taxonomic turnover of the microbial community within 30-cm depth. Such high turnover may be a common feature of vertical sediment profiles and has been reported for bacterial taxa in coastal marine sediments [42], for marine Archaea and Bacteria [43], and for freshwater Archaea [21]. Previous studies of freshwater sediments that reported moderate species turnover were restricted to low-resolution methods [15, 21, 44]. Our study differed from previous efforts in that most studies have focused on either Bacteria or Archaea and not on all three domains simultaneously, and none of the previous marine or freshwater studies have partitioned *β*-diversity into richness and replacement components. The former allowed us to examine whole-community patterns and potential interactions, and the latter allowed to distinguish taxonomic changes that result from microbial activity from those related to sediment burial. We found the microbial community to be clearly delineated into three distinct clusters, each spanning multiple sampling layers (details in Additional file 7). Based on the significant contribution of taxonomic replacement (Table 2) and measurable microbial production in the upper two clusters (clusters a, b; Fig. 1), we term this part of the sediment the “replacement horizon” (Fig. 3). We term the lower layers, comprising the deepest cluster (cluster c; Fig. 1) the “depauperate horizon,” based on the importance of taxonomic richness (as opposed to turnover) in structuring the community and based on the constancy of most sediment biogeochemical parameters at these depths (Fig. 3, Additional file 7). Both horizons are discussed in more detail below.

**The replacement horizon** We define the replacement horizon (Fig. 3) as comprising the two microbial community clusters in which taxonomic turnover was most pronounced (a, b in Fig. 1). This horizon is further subdivided by the sulfate-methane transition into what we term “redox-stratified” and “transition” zones (Information Box, Additional file 7). Bacterial activity was highest in the redox-stratified zone, where most of the settled OM is readily available. Sulfate was depleted below 5-cm depth, and therefore, most redox processes will take place above. The majority of freshwater sediment studies examine this zone in great detail (e.g., [4, 5, 10]), including the identification of redox processes at the millimeter scale [45, 46]. Active decomposition leads to high prokaryotic cell numbers close to the sediment surface [5, 25, 47] and decreasing abundance with depth [15]. We also observed an initial loss of many taxa in the highly active oxycline—indicated in our data by an outlier to the richness community component (Additional file 3)—which may have been intensified by active grazing by ciliates and copepods. Important methane oxidation processes occur in the transition zone (below the sulfate-methane transition)(cluster b, Additional file 7) [48]. At 10 cm, the CO_2_ minimum could indicate the start of hydrogenotrophic methanogenesis (Fig. 1) as previously described for profundal Lake Stechlin sediments [49]. The archaeal contribution rose constantly in the transition zone, while Bacteria and Eukaryota decreased and the general activity measures declined rapidly.

According to DNA concentrations, more than 85% of settled organisms were decomposed within this replacement horizon, which spanned approximately 60 years. The decay of DNA, in combination with an enrichment of fulvic acids with increasing depth (FI values), confirms the assumption that DNA can serve as a proxy for the buried OM. We conclude that there is a gradient of OM quality in addition to a gradient of electron acceptors in the replacement horizon. This may facilitate the stratification of active microbial taxa with depth because OM can simultaneously serve as an electron donor and acceptor [50, 51]. OM quality can also modulate microbial redox processes [52], with apparent consequences for carbon turnover rates [36, 51].

**The depauperate horizon** The depauperate horizon (Fig. 3) was characterized by high concentrations of methane (CH_4_) and CO_2_, a dominance of Archaea, and low diversity compared to the replacement horizon. Microbial community composition was more nested, with a 9% relative nestedness here compared to only 2% in the replacement horizon. By entering this horizon, the DNA:RNA ratio doubled and Archaea replaced Bacteria as the dominant microorganisms. We believe this reflects an increase in the number of microbes entering a stationary state below this depth, where cell maintenance predominates over cell synthesis due to the low availability of terminal electron acceptors. This is analogous to what has been suggested for cells in low-energy marine environments in the deep sub-seafloor sediment [27, 30, 53]. The variability in community composition was very low across replicates in the depauperate horizon. The nestedness suggests the gradual disappearance of taxa with burying age and a richness component of turnover steadily increasing to more than 20% (Table 2). If the richness component was to further increase in a linear manner, it would be the sole factor structuring the community composition deeper than 1 m (the total sediment depth of Lake Stechlin is 6 m). It is intuitive that the richness component may be a function of the burying time and that it represents the fading signal of preserved organisms. It remains unclear why it does not follow an exponential decay function analogous to that for DNA.

**Potential causes of the high taxonomic replacement** Many “present” parameters changed rapidly with depth, particularly in the replacement horizon (e.g., DNA, FI, BPP, electron acceptors), and this was a likely driver of the high degree of taxonomic turnover. Several mechanisms could be responsible for these patterns, namely cellular turnover and random appearances. In cellular turnover, taxonomic replacement is potentially caused by cell synthesis, lysis, and recycling of dormant cells, which are assumed to be high in sediments [54, 55], particularly viral lysis [56, 57]. We found indications for cellular recycling caused by the predatory Bacteriovoracaceae (cf. [58]), which was one of the structuring bacterial lineages identified in Additional file 4. Another potential mechanism—one that may be the most important in the depauperate horizon—is differential cell replication. The resources for cell maintenance and growth should depend on cell size and complexity. This means that small cells, such as nano-Archaea (e.g., Candidatus Parvarchaeum), should have a selective advantage because they can continue to grow under conditions in which larger cells must switch to cell maintenance. This could be one explanation for the observed drop in evenness in the depauperate horizon.

In the mechanism of random appearances, the appearance of taxa may be due to the disappearance of others. Because high-throughput sequencing methods produce relative (rather than absolute) data, it may superimpose proportions over quantities. For example, the initial decay of eukaryotes may have opened a niche for previously hidden rare taxa. Further, if there was no growth in the sediment, lineages that are potentially better suited for long-term survival than others would appear, such as spore-forming Bacteria (Firmicutes). However, we (and others: [27, 34]) did not observe an enrichment of this lineage with depth. In addition, the use of replicate cores in combination with our conservative stripping (see the “Methods” section) should have removed most of the random effects. The mostly constant cell numbers with small local maxima and the observed shifts in the evenness support a non-random stratification of sediment communities including cell replication. While the cellular reproduction probably approaches stagnation for most microbes in the depauperate horizon, the slowly shifting redox conditions across seasons and years may be conducive to colonization of the replacement horizon by different niche specialists. The low sedimentation rate of Lake Stechlin (ca. 2 mm per year, as determined by ^137^Cs dating at the Federal Office for Radiation Protection, Berlin [courtesy of U.-K. Schkade], or 0.4–2.1 g m ^−2^ d ^−1^, as determined by sediment traps [59]) may mitigate this stratification, and the scale of the horizons may differ in systems with higher or lower sedimentation rates.

### Burial processes and microbial activities

The microbial sediment community appeared to be highly indigenous, and yet the constant arrival of sinking OM could bury the microbial community. Indeed, buried DNA and organisms preserve historical plankton communities that can be indicative of past conditions of the lake ecosystem [34, 60, 61]. These past environmental conditions are also partly preserved as particulate matter, which is relatively conservative. We found several of these conservative “past” parameters to correlate well with the present-day communities. Although previous studies have found sediment parameters to influence community patterns in marine systems [43], the parameters were not separated into a present-past context, and microbial community turnover was not partitioned into richness and replacement components. Our results revealed that the richness component could be largely explained by the first two principal components of the past parameters. In contrast, the replacement component was not fully explained by the present parameters, indicating that sources of variation other than environmental parameters are important, such as biotic interactions. Strong biotic interactions have been identified in a vertical profile of a meromictic lake with a comparable chain of redox processes as they occur in sediments [62]. Deep sediment layers may offer low-energy niches that favor a large variety of syntrophic microorganisms [63, 64]. The Dehalococcoidales (Chloroflexi [65, 66]) and the Miscellaneous Crenarchaeotic Group (MCG, [67, 68]) are promising candidates for such hybrid forms of energy harvesting and were among the most influential lineages in our data set (Additional file 4). The MCG co-occurred with Dehalococcoidales, similar to what was found in methane hydrate-bearing sediment in Lake Baikal at >1500-m water depth [69]. Another indication that biotic interactions are important in Lake Stechlin sediments is the appearance of the Candidatus Parvarchaeum as a structuring lineage (Fig. 3). This lineage can exhibit cell-to-cell coupling that allows for thermodynamic processes that would otherwise not be possible [70].

### Archaea in freshwater sediments

The fact that Archaea can be very numerous in freshwater sediments and even dominate microbial communities is a rather new discovery, and data from comparative studies are lacking. Their recovery rate in relation to bacterial sequences or cell numbers varies between 3–12% ([71], cells), 5–18% ([21], qPCR), and 14–96% ([14], qPCR), depending on the lake, sampled sediment horizons, and methods employed. In most cases, only surface sediment samples have been considered (e.g., [72], 1% of cells), and the few studies involving vertical profiling to date are ambiguous in finding an archaeal depth gradient. Our results and those from cell counts from Lake Biwa (Japan) of [71] suggest an increase in the proportion of Archaea with sediment depth. However, the results obtained by quantitative PCR for Lake Taihu (China; [14]) and Lake Pavin (France; [21]) did not report such a relationship. Archaea have, on average, compact genomes [73] and a lower ribosomal copy number than Bacteria [74], which may lead to underestimates of archaeal abundance. Similar to our results, [21] found three sequential depth clusters in the archaeal community structure within the first 40 cm, defining an intermediate layer between 4 and 12 cm. Next to well-described methanogenic Archaea, we mainly recovered archaeal lineages with no clear functional assignment thus far (similar to [21]), i.e., primarily the MCG (potentially methanogenic, [68]) and the Marine Benthic Group D (MBG-D). Both groups are among the most numerous Archaea in the marine sub-seafloor, and they are thought to metabolize detrital proteins ([20], discussed in more detail below). Interestingly, we also identified a MBG-B as structuring OTU for the transition zone (Fig. 3), a group which was recently described as eukaryotic progenitor from a hydrothermal vent field (Lokiarchaeota, [75]). Several MCG OTUs belonged to the top structuring taxa. MCG was recently named as Bathyarchaeota by [76] for its deep-branching phylogeny and its occurrence in deep subsurface environments—environmental conditions that our cores (30-m water depth and 30-cm length) did not meet.

Our results suggest that the specific niche adaptation of these microbes is not necessarily related or restricted to the deep biosphere but rather to a cellular state of “low activity” [77]. In this context, it is interesting that single MCG OTU sometimes dominated the community in the deep horizons (up to 34% in core D at 26–30 cm), resulting in a reduced overall evenness and a shift of the residual taxa to the rare biosphere, contrasting the potential random effects as discussed above. Another intriguing observation is the considerable overlap of archaeal and partially bacterial lineages between our study and deep-sea environments. Consequently, typical marine lineages (e.g., Archaea in Additional file 4: MGI, MCG [Bathyarchaeota], MHVG, DHVEG-1, DHVEG-6 [Woesearchaeota], DSEG, MBG-A, MBG-B [Lokiarchaeota], MBG-D, MBG-E) are not as “marine” or as “deep-sea” as previously thought. Given the high cost of deep-sea research [30], freshwater sediments might literally pose a row-boat alternative for research questions targeting these “remote” and “extremophile” microorganisms.

### Study limitations and perspectives

When we look at systems that sequester carbon, it is important to keep relic DNA in mind. Relic DNA is defined as DNA residuals that remain in the system after cell death. Its presence can inflate richness and misrepresent relative abundances in some types of soils when analyzed with DNA metabarcoding [78]. The very few aquatic studies that investigated relic DNA reported large amounts of extracellular DNA in marine and freshwater sediments, with fragment sizes of up to 10 kb, but with low amplification success [79, 80]. The degradation of extracellular DNA varies widely in different environments and is dependent on the amount of OM and organic clay fractions present [81]. The low RNA content in the deeper layers in our study might indicate the presence of relic DNA; however, cell numbers in these deeper layers (>1.5 × 10^9^ cells ml ^−1^) were similar to those in upper layers (1.9–2.4 × 10^9^ cells ml ^−1^). The high DNA:RNA ratio may therefore result from an abundance of dormant and potentially dying cells [54], rather than extracellular relic DNA. In our lake, the extractable DNA seems to be rapidly decaying (similar to the sequences from eukaryotes as potential progenitors of relic DNA), which points to a short-lived fate of relic DNA in Lake Stechlin. We also found no evidence for fragmented DNA in our sediment samples that would indicate the presence of larger quantities of extracellular DNA (Additional file 8). Relic DNA would have caused an overestimate of the richness, in particular, the deeper layers would appear more species rich than they are. The use of a single SSU primer pair in our study was also a compromise and underestimates the richness of metazoan groups [37, 82], which affects the zooplankton OTUs that we found in the upper layers. This means that the richness component of *β*-diversity may be more important as a result, leading to a narrower transition zone and greater differences between the horizons. On the other hand, our study was also limited by resolution, since our pyrosequencing efforts could not adequately analyze the rare biosphere, in which we would suspect most signals from relic DNA. Future studies with higher sequencing depths and several group-specific primer pairs will be able to follow the fate of relic DNA in more details as, e.g., of Eukaryota.

## Conclusions

Our results indicate that the sediments of Lake Stechlin are a steady-state and chemostat-like environment with a highly stratified indigenous microbial community. Sediments were not a one-way system for the burial of organic matter and were not composed purely of redox-active taxa. We conclude that both processes take place alongside a vertical gradient of electron acceptors and decomposing OM of decreasing quality with depth. The microbial community was structured into distinct groups, and both the microbial community and the sediment parameters could be divided into components relevant for burial and past conditions as well as for recent carbon turnover processes and their context data. Biotic interactions are likely to play an important role, and we were able to identify important sediment taxa for each horizon. We put a spotlight on the largely unexplored freshwater sediments and confirmed earlier findings that were previously described only for marine sediments, such as the importance of marine archaeal lineages and the introduction of a depauperation zone in which the burial process becomes increasingly important.

## Methods

### Sampling site and sampling procedures

Lake Stechlin (latitude 53° 10 N, longitude 13° 02 E) is a dimictic oligo-mesotrophic lake (maximum depth 69.5 m; area 4.23 km^2^) in northern Germany that has been the subject of more than 55 years of research [83]. Sediment cores were extracted from the southern bay of the lake. Four adjacent sites were sampled to account for spatial heterogeneity in the sediment (sites A–D, Additional file 9). Pore water was collected by four in situ dialysis samplers, so-called peepers [84], which were deployed for 14 days using a frame (1 m^2^). Shortly before retrieving the peepers, four sediment cores were taken from each site with Perspex tubes (inner diameter 6 or 9 cm; length 60 cm) using gravity corers (UWITEC™, Mondsee, Austria) at 30-m water depth (aphotic depth) on two subsequent days (March 26 and 28, 2012; peepers were retrieved on April 1, 2012). Two sediment cores (6-cm diameter) were stored in the dark at 4 °C until oxygen penetration depth was measured within the next 4 h (see below). The sediments of the 9-cm cores were sliced directly into 1-cm layers for the uppermost 10 cm and then in 4-cm layers for sediment depths of 10–30 cm. One core was used for the analysis of the total sediment, and the other was used for pore water, gas, and microbial analyses (see below). In May 2014, 24 additional cores were taken to determine the age-depth correlation using the cesium 137 technique.

### Maximum oxygen penetration depth

Two initial cores were carefully transferred into 20-cm short cores without disturbing the sediment surface. The short cores were kept cool (4 °C) until measurements were taken. Oxygen microprofiles were performed using two Clark-type microelectrodes (OX50 oxygen microsensors, Unisense, Aarhus, Denmark) with a 50- *μ*m glass tip. SensorTracePro 2.0 software (Unisense) was used for data storage. The electrodes were calibrated by two-point calibration. For each core, we measured at least four profiles. The sediment-water interface was defined as the point where the oxygen depletion shifted from linear to non-linear [85].

### Pore water analysis

The sampled sediment horizons were centrifuged (13,250*g*for 10 min) to retrieve pore water (filtered through rinsed 0.45- *μ*m cellulose acetate membranes, Roth, Germany) for immediate analysis of the dissolved organic carbon (DOC) and FI. DOC was measured as non-purgeable organic carbon with an organic carbon analyzer (multi N/C 3100, Analytic Jena AG, Jena, Germany). FI was measured following the protocol of [38]. Peeper samples were analyzed for concentrations of SRP and ammonium (NH$_{4}^{+}$), dissolved iron (Fe ^2+/3+^), manganese (Mn ^2+^), chloride (Cl ^−^), nitrate (NO$_{3}^{-}$), and sulfate (SO$_{4}^{2-}$), following DIN EN ISO 10304-1. SRP and NH$_{4}^{+}$ were photometrically determined using segmented flow analysis (SFA, Skalar Sanplus, Skalar Analytical B.V., De Breda, Netherlands). Dissolved iron and manganese levels were determined by AAS (PerkinElmer 3300, Rodgau-Juegesheim, Germany), and analyses of the dissolved anions nitrate and sulfate were conducted by ion chromatography (IC, Shimadzu Corporation, Japan).

### Total sediment analysis

Sediment water content was analyzed by drying at 85 °C until mass was constant. A subsample was used to determine the organic matter content (4 h at 550 °C) of the sediment. The metal concentrations were determined by ICP-OES (iCAP 6000, Thermo Fisher Scientific, Dreieich, Germany) after aqua regia digestion in a microwave oven (Gigatherm, Grub, Switzerland), and total phosphorus (TP) was determined spectrophotometrically by CARY 1E (Varian Deutschland GmbH, Darmstadt, Germany) after H_2_SO_4_/H_2_O_2_ digestion (150 ^∘^C, 16 h). CNHS content was determined using aliquots of dried matter in a vario EL system (Elementar Analysensysteme GmbH, Hanau, Germany).

### Gas chromatography

From each depth, 2 ml of sediment was transferred into 10-ml vials filled with 4 ml of distilled water. Samples were fixed with mercury chloride (final conc. 200 mg l ^−1^), sealed, and stored in the dark at 4 °C until analysis. Concentrations of CO_2_, CH_4_, and N_2_O were measured by gas chromatography (Shimadzu GC-14B, Kyoto, Japan) using the headspace technique described in [86].

### Bacterial protein production

Bacterial biomass production was determined via ^14^C leucine incorporation at in situ temperature under anoxic conditions [87] using a modified protocol [88]. Five hundred microliters of sediment was diluted 1:1 with sterile filtered supernatant water and incubated with ^14^C-leucine (Hartmann Analytics, Braunschweig, Germany; specific activity 306 mCi mmol^−1^, diluted with cold L-leucine to a final concentration of 50 μmol l^−1^). Incubations were stopped after 1 h, extracted, and measured in a liquid scintillation analyzer (TriCarb 2810 TR, PerkinElmer Inc., Germany). Disintegrations per minute were converted to pmol leucine ml ^−1^ day ^−1^, assuming a twofold intracellular isotope dilution [89, 90].

### Cell counting

Sediment subsamples for cell counting were immediately fixed with ethanol (50% *v*/*v*final concentration). Prior to analysis, samples were shaken for 1 h at 700 rpm on a thermoshaker and were sonicated three times for 20 s at 5–6 W (Branson Sonifier 150, Danbury, USA). Cells were stained with a SYBR Gold staining solution diluted to 1:1000 (Molecular Probes, Eugene, USA) and were counted with an epifluorescence microscope (Zeiss, Axio Imager. Z1, Jena, Germany).

### Nucleic acid extraction and sequencing

To determine the DNA:RNA ratio (as part of the present parameters, Additional file 10), we extracted total nucleic acids using a phenol-chloroform protocol from 200–400 *μ*l sediment, as described by [91]. The DNA:RNA ratio was measured via fluorometry using selectively binding dyes (broad range dsDNA and broad range RNA assay Kit, Life technologies, Darmstadt, Germany) developed for the Qubit 2.0 (Life technologies, Darmstadt, Germany). A second extraction served as template for the sequencing and determination of the total DNA content. A defined sediment subsample (350 *μ*l) from each depth was lyophilized prior to DNA extraction. We used the “Alternative Protocol for Maximum DNA yields” of the UltraClean®; Soil DNA Isolation Kit (MoBio Laboratories Inc., Carlsbad, USA). The quality of the DNA and the presence of putative environmental (small) DNA in 12 representative samples from 12 depths were verified with a microgel electrophoresis system (DNA High Sensitivity Kit, Bioanalyzer, Agilent, USA, see Additional file 8). A total of 5–20 ng of DNA, as measured by NanoPhotometer P300 (Implen, Schatzbogen, Germany), served as the template for PCR amplification (Herculase II system, Life Technologies) using a single universal primer system (926F, 1392R, [92]) targeting the SSU V6-V8 region. The primer pair employed is one important feature of our study in that it detects similar to the primer of [93] all three microbial domains (Archaea, Bacteria, and Eukaryota) in freshwater systems [37, 62, 94]. The variability of the V6–V8 is sufficiently high for all three domains [95–97]. PCR products were purified with AMPure Beads (Beckmann Coulter, Brea, USA) and quantified and pooled using a PicoGreen assay (Life Technologies, Carlsbad, USA). High-throughput sequencing was performed in a Roche 454 GS Junior benchtop sequencer (Hoffmann-La Roche, Basel, Switzerland) at the Berlin Center for Genomics in Biodiversity Research.

### Data processing

Four hundred fifty-four sequencing data were processed using Mothur (version 1.33.0) following the guidelines of the Mothur SOP (http://www.mothur.org/wiki/454_SOP, accessed 02/2014) with the following modifications: (i) for quality trimming, we used a sliding window approach with a relaxed threshold (window size 50, quality cutoff 27), and (ii) the alignment step used SINA (version 1.2.11; [98]) against the SILVA v.111 non-redundant SSU reference database. A total of 396,000 reads (49% of 802,202 raw sequences) were retained. The sequences were clustered into OTUs at 97% sequence similarity (Additional file 10). A representative sequence from each OTU was used for taxonomic classification with the least common ancestor method in SINA, using 0.7 as a setting for minimum similarity as well as for lca-quorum. The classified OTU abundance matrix served as the basis for all subsequent statistical analyses (Additional file 11). The percentage of sequences with low similarity (< 93%) to the next reference sequence was determined by submitting the FASTA files to SILVA NGS [99].

### Statistics

All measured environmental parameters were compiled in a matrix and imported into R (http://cran.r-project.org/, version 3.2.2; Additional file 12). We replaced two outliers (FI: replicate 4 cm, total phosphorous: replicate 14 cm) with the mean values of the three other sediment cores. Similarly, the 30 cm peeper data from replicate core B were missing and replaced by the mean of the residual replicates. For statistical analysis, the relative proportions of Archaea, Bacteria, and Eukaryota were arc-sin transformed. For the multiple regression analysis on the declining DNA concentrations, we removed one value (replicate 3, 22 cm) to meet the normal distribution criteria of the residuals. Sufficient normal distribution was confirmed by a QQ plot and Shapiro-Wilks test, *p*=0.183; Cook’s distance was not violated in any case. We categorized the environmental parameters into present (CH_4_, CO_2_, DOC, BPP, SRP, NH$_{4}^{+}$, SO$_{4}^{2-}$, Cl ^−^, Fe ^2+/3+^, Mn ^2+^, FI, and RNA:DNA) and past (TC, TN, dry-weight, TP, TS, TH, Al, As, Ca, Cu, Fe, Mg, Mn, Pb, Ti, and Zn) (see Table 1) and assessed the sample variation for each subset by a centered, scaled principal component analysis (PCA, see Additional file 10). The resulting most explanatory PCA axes, which explained 54% (present parameters) and 41% (past parameters) of the sample variation, were used in the community statistics (see below). DNA and cell numbers were not categorized due to their ambiguous nature; Al was used as a substitute for Mg and Ti due to their high degree of correlation (*r*>0.95); N_2_O, NO$_{3}^{-}$, Cd, and Co were excluded due to their very low values, i.e., near or below the detection limit in all the samples.

#### Community statistics

Random effects were initially excluded from the OTU matrix by removing all OTUs present in only one sample, regardless of the number of reads. The random effects are expected to be very high in sediments due to the burial of random organic matter (e.g., caused by bird droppings, tourist activities, rainfall), and thus, a large number of rare taxa are expected. This reduced the number of OTUs from 29,228 to 9581 but did not influence the sample distances (Mantel test with Hellinger distances: *r*=0.993,*p*<0.001). Diversity indices were calculated using the vegan package [100] for R, with a community matrix that was rarefied to the lowest number of reads (642) present in a sample. The rarefied matrix was highly correlated to the initial matrix (Mantel test with Hellinger distances: *r*=0.923,*p*<0.001). Nonmetric multidimensional scaling (NMDS) and Mantel tests were calculated based on Hellinger-transformed rarefied OTU matrix with Euclidean distances. The PCA scores from the past and present parameters (see Table 1) were fitted into the NMDS, and their correlation with the underlying distance matrix was tested with a Mantel test. Additionally, we calculated the weighted UniFrac distances with the R package GUniFrac [101] and corresponding phylogenetic distances were based on a maximum likelihood tree calculated with FastTree 2.1 [102]. The weighted UniFrac distances were based on proportional data from the random effects reduced community matrix (9581 OTUs) and were projected as NMDS or MDS. We include the same statistics as in Fig. 4 (Additional file 6). Moreover, we used a fuzzy set ordination [103] to test for the influence of the past and present parameters on the separated richness and replacement community components. For this, we partitioned *β*-diversity into richness and replacement components using indices from the Jaccard family, following [104] and the functions provided by [32]. In order to identify general vertical patterns, we used a sum table to increase the resolution and thus avoid an artificial increase in turnover versus the richness/nestedness structure due to sampling effects. The sum table was generated by summing up 2000 sequences per depth, if applicable. The final sum table was rarefied to the lowest number of sequences in the depth profile (5987 reads). Hill numbers and rarefaction curves of the Hill numbers were calculated with the iNEXT package [105]. The cluster analysis (UPGMA clustering based on Kulczynski distance, Fig. 1) was also done on the sum table. The depth-dependent nestedness [31], richness component, replacement component, species contribution to beta diversity (SCBD), and LCBD were calculated as described in [32] including significance tests. We note that the nestedness index [31] is dependent on the sample size, and so we refer to it as “relative nestedness”.

## Additional files

Additional file 1 Krona chart of recovered sediment taxa. Browsable Krona chart ([107], S1.html, please use an internet browser with network access to open the file) of all taxa based on the median occurrence of OTUs for each depth replicate and classified against the SILVA reference database (www.arb-silva.de, version 111). (HTML 114 kb)

Additional file 2Figure: detailed depth profiles of individual cores. Additional detailed depth profiles of individual core (A,B,C,D) variables of Lake Stechlin at 30-m depth. Units: w.c.(water content) [%]; C [%]; N [%]; S [%];H [%]; Ca [mg g ^−1^ dry weight]; Mg [mg g ^−1^ dry weight]; NO$_{3}^{-}$ [mg l ^−1^]; SO$_{4}^{2-}$ [mg l ^−1^]; Fe ^2+/3+^ [mg l ^−1^]; Mn ^2+^ [mg l ^−1^]; Al [mg g ^−1^ dry weight]; Cd [mg g ^−1^ dry weight]; Co [mg g ^−1^ dry weight]; Cr [mg g ^−1^ dry weight]; Cu [mg g ^−1^ dry weight]; Mn [mg g ^−1^ dry weight]; Ni [mg g ^−1^ dry weight]; Ti [mg g ^−1^ dry weight]; Zn [mg g ^−1^ dry weight]. See [108] for comparison with previous data. (PDF 91 kb)

Additional file 3 Figure: richness component vs. depth. Increasing richness component with increasing depth. The first cm is an outlier of the observed linearity. (PDF 15 kb)

Additional file 4 Figure: taxonomic composition of the most structuring taxa. Hierarchical taxonomic presentation of the most structuring taxa (SCBD), i.e., all OTUs that account for more than 5 per mill of the total *β*-diversity (see inlet to the left). The pie chart is color coded according to the three domains: Bacteria (red), Archaea (green), and Eukaryota (blue). (PDF 197 kb)

Additional file 5 Figure: sediment DNA as a function of present taxonomic signals. Multiple linear regression on the sediment DNA content as a function of the occurrence of Eukaryota (75.6% of the variation) together with Bacteria (10.0% of the variation; model: *R* ^2^=0.856, *p*<0.001). (PDF 10 kb)

Additional file 6 Figure: UniFrac ordinations. Left panel - A nonmetric multidemsional scaling (analogous to Fig. 4 b) of all the samples based weighted UniFrac distances. This was also reflected in the distance between the surface and deep sediments on axis 1 (adonis: *R* ^2^=0.520,*p*<0.001). We were able to significantly recover the three depth zones (adonis: *R* ^2^=0.601,*p*<0.001). The overall community structure was correlated with both present (Mantel correlation: *r*=0.512,*p*<0.001) and past (*r*=0.333,*p*<0.001) parameters, which were nearly orthogonal in ordination. Right panel—a metric multidimensional scaling (principal coordinate analysis) of the UniFrac distance matrix that is displayed in Fig. 3 b, with the corresponding proportional eigenvalues for each axis. The curved shape may point to an ordination artifact. (PDF 27 kb)

Additional file 7 Text: sediment zonation according to taxonomic clustering, *β*-partitioning, and context data. (PDF 14 kb)

Additional file 8 Figure: DNA size distribution after extraction. DNA microgel electrophoresis (Experion, BioRad) from a random subset of samples from various sediment depths, showing the absence of small environmental DNA (<1 kb). (PDF 395 kb)

Additional file 9 Figure: sampling locations within Lake Stechlin, Germany. Depth map of Lake Stechlin (Germany) and the four replicate sampling sites (A,B,C,D) in the South-West bay with the corresponding oxygen penetration depth in cm (pink bars). (PDF 389 kb)

Additional file 10 Figure: principal component analysis of environmental parameters. Principal component analysis defining the “present” (left panel) and “past” (right panel) parameters. The samples are color coded according to the three depth clusters (a–c). (PDF 52 kb)

Additional file 11 Dataset: classified OTU table. Tab-separated text file that compiles the complete OTU table with all samples, read counts, representative FASTA sequences, and classifications. (CSV 1920 kb)

Additional file 12 Dataset: environmental parameters. Tab-separated text file that compiles all environmental parameters. (CSV 1570 kb)

## References

[1] Tranvik LJ, Downing JA, Cotner JB, Loiselle SA, Striegl RG, Ballatore TJ, Dillon P, Finlay K, Fortino K, Knoll LB (2009). Lakes and reservoirs as regulators of carbon cycling and climate. Limnol Oceanography.

[2] Dean WE, Gorham E (1998). Magnitude and signifigance of carbon burial in lakes, reservoirs, and peatlands. Geology.

[3] Bastviken D, Tranvik LJ, Downing JA, Crill PM, Enrich-Prast A (2011). Freshwater methane emissions offset the continental carbon sink. Science.

[4] Fischer H, Sachse A, Steinberg CEW, Pusch M (2002). Differential retention and utilization of dissolved organic carbon by bacteria in river sediments. Limnol Oceanography.

[5] Haglund AL, Lantz P, Törnblom E, Tranvik L (2003). Depth distribution of active bacteria and bacterial activity in lake sediment. FEMS Microbiol Ecol.

[6] Nealson KH (1997). Sediment bacteria: who’s there, what are they doing, and what’s new?. Annu Rev Earth Planet Sci.

[7] Megonigal JP, Hines ME, Visscher PT (2004). Anaerobic metabolism: linkages to trace gases and aerobic processes. Biogeochemistry.

[8] Maerki M, Muller B, Dinkel C, Wehrli B (2009). Mineralization pathways in lake sediments with different oxygen and organic carbon supply. Limnol Oceanography.

[9] Melton ED, Swanner ED, Behrens S, Schmidt C, Kappler A (2014). The interplay of microbially mediated and abiotic reactions in the biogeochemical Fe cycle. Nat Rev Microbiol.

[10] Frindte K, Allgaier M, Grossart HP, Eckert W (2015). Microbial response to experimentally controlled redox transitions at the sediment water interface. PLOS ONE.

[11] Lloyd KG, Schreiber L, Petersen DG, Kjeldsen KU, Lever MA, Steen AD, Stepanauskas R, Richter M, Kleindienst S, Lenk S, Schramm A, Jørgensen BB (2013). Predominant archaea in marine sediments degrade detrital proteins. Nature.

[12] Miskin I, Rhodes G, Lawlor K, Saunders JR, Pickup RW (1998). Bacteria in post-glacial freshwater sediments. Microbiology.

[13] Li S, Xiao X, Yin X, Wang F (2006). Bacterial community along a historic lake sediment core of Ardley Island, west Antarctica. Extremophiles.

[14] Ye W, Liu X, Lin S, Tan J, Pan J, Li D, Yang H (2009). The vertical distribution of bacterial and archaeal communities in the water and sediment of Lake Taihu. FEMS Microbiol Ecol.

[15] Vuillemin A, Ariztegui D (2013). Geomicrobiological investigations in subsaline maar lake sediments over the last 1500 years. Quaternary Sci Rev.

[16] Xiong W, Xie P, Wang S, Niu Y, Yang X, Chen W (2015). Sources of organic matter affect depth-related microbial community composition in sediments of Lake Erhai, Southwest China. J Limnol.

[17] Spring S, Schulze R, Overmann J, Schleifer KH (2000). Identification and characterization of ecologically significant prokaryotes in the sediment of freshwater lakes: molecular and cultivation studies. FEMS Microbiol Rev.

[18] Briée C, Moreira D, López-García P (2007). Archaeal and bacterial community composition of sediment and plankton from a suboxic freshwater pond. Res Microbiol.

[19] Lipp JS, Morono Y, Inagaki F, Hinrichs KU (2008). Significant contribution of Archaea to extant biomass in marine subsurface sediments. Nature.

[20] Lloyd KG, May MK, Kevorkian RT, Steen AD (2013). Meta-analysis of quantification methods shows that archaea and bacteria have similar abundances in the subseafloor. Appl Environ Microbiol.

[21] Borrel G, Lehours AC, Crouzet O, Jézéquel D, Rockne K, Kulczak A, Duffaud E, Joblin K, Fonty G (2012). Stratification of Archaea in the deep sediments of a freshwater meromictic lake: vertical shift from methanogenic to uncultured archaeal lineages. PLoS ONE.

[22] Zhang J, Yang Y, Zhao L, Li Y, Xie S, Liu Y (2015). Distribution of sediment bacterial and archaeal communities in plateau freshwater lakes. Appl Microbiol Biotechnol.

[23] Rodrigues T, Catão E, Bustamante MMC, Quirino BF, Kruger RH, Kyaw CM (2014). Seasonal effects in a lake sediment archaeal community of the Brazilian Savanna. Archaea.

[24] Gächter R, Meyer JS, Mares A (1988). Contribution of bacteria to release and fixation of phosphorus in lake sediments. Limnol Oceanography.

[25] Chan OC, Claus P, Casper P, Ulrich A, Lueders T, Conrad R (2005). Vertical distribution of structure and function of the methanogenic archaeal community in Lake Dagow sediment. Environ Microbiol.

[26] Polymenakou PN, Fragkioudaki G, Tselepides A (2007). Bacterial and organic matter distribution in the sediments of the Thracian Sea (NE Aegean Sea). Cont Shelf Res.

[27] Lomstein BA, Langerhuus AT, D’Hondt S, Jørgensen BB, Spivack AJ (2012). Endospore abundance, microbial growth and necromass turnover in deep sub-seafloor sediment. Nature.

[28] Xie S, Lipp JS, Wegener G, Ferdelman TG, Hinrichs K. -u. (2013). Turnover of microbial lipids in the deep biosphere and growth of benthic archaeal populations,. Proc Nat Acad Sci USA.

[29] Rothfuss F, Bender M, Conrad R (1997). Survival and activity of bacteria in a deep, aged lake sediment (Lake Constance). Microbial Ecol.

[30] Hoehler TM, Jørgensen BB (2013). Microbial life under extreme energy limitation. Nat Rev Microbiol.

[31] Baselga A (2010). Partitioning the turnover and nestedness components of beta diversity. Global Ecol Biogeography.

[32] Legendre P (2014). Interpreting the replacement and richness difference components of beta diversity. Global Ecol Biogeography.

[33] Corinaldesi C, Beolchini F, Dell’Anno A (2008). Damage and degradation rates of extracellular DNA in marine sediments: implications for the preservation of gene sequences. Mol Ecol.

[34] Wunderlin T, Corella JP, Junier T, Bueche M, Loizeau JL, Girardclos S, Junier P (2014). Endospore-forming bacteria as new proxies to assess impact of eutrophication in Lake Geneva (Switzerland-France). Aquat Sci.

[35] Meyers PA, Ishiwatari R (1993). Lacustrine organic geochemistry—an overview of indicators of organic-matter sources and diagenesis in lake-sediments. Org Geochem.

[36] Arndt S, Jørgensen BB, LaRowe DE, Middelburg JJ, Pancost RD, Regnier P (2013). Quantifying the degradation of organic matter in marine sediments: a review and synthesis. Earth-science Rev.

[37] Wurzbacher C, Attermeyer K, Kettner MT, Flintrop C, Warthmann N, Hilt S, Grossart HP, Monaghan MT. Metabarcoding of unfractionated water samples relates phyto-, zoo-and bacterioplankton dynamics and reveals a single-taxon bacterial bloom. bioRxiv. 2016:058628. doi:10.1101/058628. 10.1111/1758-2229.12540 28429584

[38] McKnight DM, Boyer EW, Westerhoff PK, Doran PT, Kulbe T, Andersen DT (2001). Spectrofluorometric characterization of dissolved organic matter for indication of precursor organic material and aromaticity. Limnol Oceanography.

[39] Chao A, Gotelli NJ, Hsieh T, Sander EL, Ma K, Colwell RK, Ellison AM (2014). Rarefaction and extrapolation with Hill numbers: a framework for sampling and estimation in species diversity studies. Ecol Monographs.

[40] Comte J, Lindström ES, Eiler A, Langenheder S (2014). Can marine bacteria be recruited from freshwater sources and the air?. ISME J.

[41] Capone DG, Kiene RP (1988). Comparison of microbial dynamics in marine and freshwater sediments: contrasts in anaerobic carbon catabolism. Limnol Oceanography.

[42] Luna GM, Corinaldesi C, Rastelli E, Danovaro R (2013). Patterns and drivers of bacterial alpha- and beta-diversity across vertical profiles from surface to subsurface sediments. Environ Microbiol Rep.

[43] Jørgensen SL, Hannisdal B, Lanzén A, Baumberger T, Flesland K, Fonseca R, Ovreås L, Steen IH, Thorseth IH, Pedersen RB, Schleper C (2012). Correlating microbial community profiles with geochemical data in highly stratified sediments from the Arctic Mid-Ocean Ridge. Proc Nat Acad Sci USA.

[44] Koizumi Y, Kojima H, Fukui M (2003). Characterization of depth-related microbial community structure in lake sediment by denaturing gradient gel electrophoresis of amplified 16S rDNA and reversely transcribed 16S rRNA fragments. FEMS Microbiol Ecol.

[45] Sass H, Cypionka H, Babenzien HD (1997). Vertical distribution of sulfate-reducing bacteria at the oxic-anoxic interface in sediments of the oligotrophic Lake Stechlin. FEMS Microbiol Ecol.

[46] Deutzmann JS, Stief P, Brandes J, Schink B (2014). Anaerobic methane oxidation coupled to denitrification is the dominant methane sink in a deep lake. Proc Nat Acad Sci USA.

[47] Thevenon F, Graham ND, Chiaradia M, Arpagaus P, Wildi W, Poté J (2011). Local to regional scale industrial heavy metal pollution recorded in sediments of large freshwater lakes in central Europe (lakes Geneva and Lucerne) over the last centuries. Sci Total Environ.

[48] Treude T, Krause S, Maltby J, Dale AW, Coffin R, Hamdan LJ (2014). Sulfate reduction and methane oxidation activity below the sulfate-methane transition zone in Alaskan Beaufort Sea continental margin sediments : implications for deep sulfur cycling. Geochimica et Cosmochimica Acta.

[49] Conrad R, Chan OC, Claus P, Casper P (2007). Characterization of methanogenic Archaea and stable isotope fractionation during methane production in the profundal sediment of an oligotrophic lake (Lake Stechlin, Germany). Limnol Oceanography.

[50] Lovley DR, Coates JD, Blunt-Harris EL, Phillips EJP, Woodward JC (1996). Humic substances as electron acceptors for microbial respiration. Nature.

[51] Torres IC, Inglett KS, Reddy KR (2011). Heterotrophic microbial activity in lake sediments: effects of organic electron donors. Biogeochemistry.

[52] Glombitza C, Stockhecke M, Schubert CJ, Vetter A, Kallmeyer J (2013). Sulfate reduction controlled by organic matter availability in deep sediment cores from the saline, alkaline Lake Van (Eastern Anatolia, Turkey). Front Microbiol.

[53] Parkes RJ, Cragg B, Roussel E, Webster G, Weightman A, Sass H (2014). A review of prokaryotic populations and processes in sub-seafloor sediments, including biosphere: geosphere interactions. Marine Geol.

[54] Luna GM, Manini E, Danovaro R (2002). Large fraction of dead and inactive bacteria in coastal marine sediments: comparison of protocols for determination and ecological significance. Appl Environ Microbiol.

[55] Danovaro R, Dell’Anno A, Corinaldesi C, Magagnini M, Noble R, Tamburini C, Weinbauer M (2008). Major viral impact on the functioning of benthic deep-sea ecosystems,. Nature.

[56] Engelhardt T, Kallmeyer J, Cypionka H, Engelen B (2014). High virus-to-cell ratios indicate ongoing production of viruses in deep subsurface sediments. ISME J.

[57] Dell’Anno A, Corinaldesi C, Danovaro R (2015). Virus decomposition provides an important contribution to benthic deep-sea ecosystem functioning. Proc Nat Acad Sci.

[58] Kandel PP, Pasternak Z, van Rijn J, Nahum O, Jurkevitch E (2014). Abundance, diversity and seasonal dynamics of predatory bacteria in aquaculture zero discharge systems. FEMS Microbiol Ecol.

[59] Fuchs A, Selmeczy GB, Kasprzak P, Padisák J, Casper P (2016). Coincidence of sedimentation peaks with diatom blooms, wind, and calcite precipitation measured in high resolution by a multi-trap. Hydrobiologia.

[60] Domaizon I, Savichtcheva O, Debroas D, Arnaud F, Villar C, Pignol C, Alric B, Perga ME (2013). DNA from lake sediments reveals the long-term dynamics and diversity of *Synechococcus* assemblages. Biogeosci Discuss.

[61] Capo E, Debroas D, Arnaud F, Domaizon I (2015). Is planktonic diversity well recorded in sedimentary DNA? Toward the reconstruction of past protistan diversity. Microb Ecol.

[62] Gies EA, Konwar KM, Thomas Beatty J, Hallam SJ (2014). Illuminating microbial dark matter in meromictic Sakinaw Lake. Appl Environ Microbiol.

[63] Adrian L, Szewzyk U, Wecke J, Görisch H (2000). Bacterial dehalorespiration with chlorinated benzenes. Nature.

[64] Ferrer M, Guazzaroni ME, Richter M, García-Salamanca A, Yarza P, Suárez-Suárez A, Solano J, Alcaide M, van Dillewijn P, Molina-Henares MA, López-Cortés N, Al-Ramahi Y, Guerrero C, Acosta A, de Eugenio LI, Martínez V, Marques S, Rojo F, Santero E, Genilloud O, Pérez-Pérez J, Rosselló-Móra R, Ramos JL (2011). Taxonomic and functional metagenomic profiling of the microbial community in the anoxic sediment of a sub-saline shallow lake (Laguna de Carrizo, Central Spain). Microb Ecol.

[65] Hiraishi A (2008). Biodiversity of dehalorespiring bacteria with special emphasis on polychlorinated biphenyl/dioxin dechlorinators. Microbes Environ.

[66] Hug LA, Castelle CJ, Wrighton KC, Thomas BC, Sharon I, Frischkorn KR, Williams KH, Tringe SG, Banfield JF (2013). Community genomic analyses constrain the distribution of metabolic traits across the Chloroflexi phylum and indicate roles in sediment carbon cycling. Microbiome.

[67] Sørensen KB, Teske A (2006). Stratified communities of active Archaea in deep marine subsurface sediments stratified communities of active Archaea in deep marine subsurface sediments. Appl Environ Microbiol.

[68] Evans PN, Parks DH, Chadwick GL, Robbins SJ, Orphan VJ, Golding SD, Tyson GW (2015). Methane metabolism in the archaeal phylum Bathyarchaeota revealed by genome-centric metagenomics. Science.

[69] Kadnikov VV, Mardanov AV, Beletsky AV, Shubenkova OV, Pogodaeva TV, Zemskaya TI, Ravin NV, Skryabin KG (2012). Microbial community structure in methane hydrate-bearing sediments of freshwater Lake Baikal. FEMS Microbiol Ecol.

[70] Baker BJ, Comolli LR, Dick GJ, Hauser LJ, Hyatt D, Dill BD, Land ML, VerBerkmoes NC, Hettich RL, Banfield JF (2010). Enigmatic, ultrasmall, uncultivated Archaea. Proc Nat Acad Sci.

[71] Koizumi Y, Takii S, Nishino M, Nakajima T (2003). Vertical distributions of sulfate-reducing bacteria and methane-producing archaea quantified by oligonucleotide probe hybridization in the profundal sediment of a mesotrophic lake. FEMS Microbiol Ecol.

[72] Schwarz JIK, Eckert W, Conrad R (2007). Community structure of Archaea and Bacteria in a profundal lake sediment Lake Kinneret (Israel). Syst Appl Microbiol.

[73] Koonin EV, Wolf YI (2008). Genomics of bacteria and archaea: the emerging dynamic view of the prokaryotic world. Nucleic Acids Res.

[74] Angly FE, Dennis PG, Skarshewski A, Vanwonterghem I, Hugenholtz P, Tyson GW (2014). CopyRighter: a rapid tool for improving the accuracy of microbial community profiles through lineage-specific gene copy number correction. Microbiome.

[75] Spang A, Saw JH, Jørgensen SL, Zaremba-Niedzwiedzka K, Martijn J, Lind AE, van Eijk R, Schleper C, Guy L, Ettema TJ (2015). Complex archaea that bridge the gap between prokaryotes and eukaryotes. Nature.

[76] Meng J, Xu J, Qin D, He Y, Xiao X, Wang F (2014). Genetic and functional properties of uncultivated MCG archaea assessed by metagenome and gene expression analyses. ISME J.

[77] Kubo K, Lloyd KG, F Biddle J, Amann R, Teske A, Knittel K (2012). Archaea of the miscellaneous crenarchaeotal group are abundant, diverse and widespread in marine sediments. ISME J.

[78] Carini P, Marsden PJ, Leff JW, Morgan EE, Strickland MS, Fierer N (2016). Relic DNA is abundant in soil and obscures estimates of soil microbial diversity. Nat Microbiol.

[79] Corinaldesi C, Danovaro R, Dell’Anno A (2005). Simultaneous recovery of extracellular and intracellular DNA suitable for molecular studies from marine sediments. Appl Environ Microbiol.

[80] Ogram A, Sayler GS, Barkay T (1987). The extraction and purification of microbial DNA from sediments. J Microbiol Methods.

[81] Cai P, Huang QY, Zhang XW (2006). Interactions of DNA with clay minerals and soil colloidal particles and protection against degradation by DNase. Environ Sci Technol.

[82] Tang CQ, Leasi F, Obertegger U, Kieneke A, Barraclough TG, Fontaneto D (2012). The widely used small subunit 18S rDNA molecule greatly underestimates true diversity in biodiversity surveys of the meiofauna. Proc Nat Acad Sci.

[83] Koschel R, Adams D (2003). An approach to understanding a temperate oligotrophic lowland lake (Lake Stechlin, Germany). Archiv fur Hydrobiologie, Special Issues Adv Limnol.

[84] Hesslein RH (1976). An in situ sampler for close interval pore water studies. Limnol Oceanography.

[85] Glud RN (2008). Oxygen dynamics of marine sediments. Marine Biol Res.

[86] Casper P, Albino MF, Adams DD (2009). Diffusive fluxes of CH4 and CO2 across the water-air interface in the eutrophic Lake Dagow, northeast Germany. Verh. Int. Verein. Limnol.

[87] Buesing N, Gessner MO (2003). Incorporation of radiolabeled leucine into protein to estimate bacterial production in plant litter, sediment, epiphytic biofilms, and water samples. Microb Ecol.

[88] Attermeyer K, Premke K, Hornick T, Hilt S, Grossart HP (2013). Ecosystem-level studies of terrestrial carbon reveal contrasting bacterial metabolism in different aquatic habitats. Ecology.

[89] Azam F, Simon M (1989). Protein content and protein synthesis rates of planktonic marine bacteria. Marine Ecol Prog Ser.

[90] Kemp PF, Sherr BF, Sherr EB, Cole JJ (1993). Leucine incorporation as a measure of biomass production by heterotrophic bacteria. Handbook of methods in aquatic microbial ecology.

[91] Nercessian O, Noyes E, Kalyuzhnaya MG, Lidstrom ME, Chistoserdova L (2005). Bacterial populations active in metabolism of C 1 compounds in the sediment of Lake Washington, a freshwater lake. Appl Environ Microbiol.

[92] Engelbrektson A, Kunin V, Wrighton KC, Zvenigorodsky N, Chen F, Ochman H, Hugenholtz P (2010). Experimental factors affecting PCR-based estimates of microbial species richness and evenness. ISME J.

[93] Parada AE, Needham DM, Fuhrman JA (2016). Every base matters: assessing small subunit rRNA primers for marine microbiomes with mock communities, time series and global field samples. Environ Microbiol..

[94] Hölker F, Wurzbacher C, Weißenborn C, Monaghan MT, Holzhauer SIJ, Premke K (2015). Microbial diversity and community respiration in freshwater sediments influenced by artificial light at night. Phil Trans R Soc London. Series B, Biol Sci.

[95] Vasileiadis S, Puglisi E, Arena M, Cappa F, Cocconcelli PS, Trevisan M (2012). Soil bacterial diversity screening using single 16S rRNA gene V regions coupled with multi-million read generating sequencing technologies. PloS one.

[96] Seedorf H, Kittelmann S, Henderson G, Janssen PH (2014). Rim-db: a taxonomic framework for community structure analysis of methanogenic archaea from the rumen and other intestinal environments. PeerJ.

[97] Hadziavdic K, Lekang K, Lanzen A, Jonassen I, Thompson EM, Troedsson C (2014). Characterization of the 18S rrna gene for designing universal eukaryote specific primers. PLoS One.

[98] Pruesse E, Peplies J, Glöckner FO (2012). SINA: accurate high throughput multiple sequence alignment of ribosomal RNA genes. Bioinformatics.

[99] Quast C, Pruesse E, Yilmaz P, Gerken J, Schweer T, Yarza P, Peplies J, Glöckner F (2013). The SILVA ribosomal RNA gene database project: improved data processing and web-based tools. Nucleic Acids Res.

[100] Oksanen J, Blanchet FG, Kindt R, Legendre P, Minchin PR, O’Hara RB, Simpson GL, Solymos P, Stevens HH, Wagner H. vegan: Community Ecology Package. R package version 2.3-5. 2016. https://CRAN.Rproject.org/package=vegan.

[101] Chen J, Bittinger K, Charlson ES, Hoffmann C, Lewis J, Wu GD, Collman RG, Bushman FD, Li H (2012). Associating microbiome composition with environmental covariates using generalized unifrac distances. Bioinformatics.

[102] Price MN, Dehal PS, Arkin AP (2010). Fasttree 2—approximately maximum-likelihood trees for large alignments. PloS one.

[103] Roberts DW (2008). Statistical analysis of multidimensional fuzzy set ordinations. Ecology.

[104] Carvalho JC, Cardoso P, Gomes P (2012). Determining the relative roles of species replacement and species richness differences in generating beta-diversity patterns. Global Ecol Biogeography.

[105] Hsieh T, Ma K, Chao A (2016). iNEXT: an R package for rarefaction and extrapolation of species diversity (Hill numbers). Methods Ecol Evol.

[106] Chiu CH, Wang YT, Walther BA, Chao A (2014). An improved nonparametric lower bound of species richness via a modified good-turing frequency formula. Biometrics.

[107] Ondov BD, Bergman NH, Phillippy AM (2011). Interactive metagenomic visualization in a web browser. BMC Bioinformatics.

