# Supplementary material for: Shifts among Eukaryota, Bacteria, and Archaea define the vertical organization of a lake sediment
Source: Microbiome. 2017 Apr 8;5:41. doi: 10.1186/s40168-017-0255-9 (PMC5385010; doi:10.1186/s40168-017-0255-9)
Supplement: Supplementary file 1 — Krona chart of recovered sediment taxa. Browsable Krona chart ([107], S1.html, please use an internet browser with network access to open the file) of all taxa based on the median occurrence of OTUs for each depth replicate and classified against the SILVA reference database (www.arb-silva.de, version 111). (HTML 114 kb) [file 40168_2017_255_MOESM1_ESM.html]

Javascript must be enabled to view this page.

members
magnitude

10\_cm
14\_cm
18\_cm
1\_cm
22\_cm
26\_cm
2\_cm
30\_cm
3\_cm
4\_cm
5\_cm
6\_cm
7\_cm
8\_cm
9\_cm

302.5387.5431330403422.5306.5474.5342321328308330315323

60.582.50.583.5624215132813.56.5

3.50.560593827.5119127.56

20.541.54930.521.59.55.51064

234.540.526.516.564.5863

234.540.526.516.564.5863

1.59.511105.542.56.531.5

1.59.511105.542.56.531.5

2.5220.5

0.5232616.511221.531.5

0.521.52616.510.5221.531.5

1.50.5

1.50.5

0.578.5453.5121

0.578.5453.5121

241.52.50.5

131.52.50.5

131.52.50.5

11

11

320.50.50.5

0.50.50.5

0.50.50.5

210.5

210.5

0.50.50.5

0.50.50.5

0.522.5222.5121

0.522.5222.5121

420.50.50.5

420.50.50.5

0.5

420.5

0.5

0.5

0.5

0.5

0.5

1.514.5875.513.521.52

0.54.51.51.510.51

10.5

10.5

1

0.5

0.5

1.5

1.5

174541311.52

3.54.52.512.5111

3.54.52.512.5111

3.54.52.512.5111

10.50.51.50.50.51

10.50.51.50.50.51

4

1

3

3

0.5

0.5

2.5

2.5

120.50.5

0.5

111.50.520.50.5

111.50.520.50.5

111.50.520.50.5

10.51.50.520.50.5

0.51

10.50.520.50.5

0.5

1.521.50.52323.512.543.5165.50.5

2.50.52.50.52.5

1.50.50.51.5

1.50.50.51.5

1.50.50.51.5

0.5

10.51

120.5

1.51920.5231043.5165.50.5

0.50.5

0.50.5

1.518.520239.543.5165.50.5

1.518.520239.543.5165.50.5

1.521.50.50.5

1.51.51.50.50.5

1.51.51.50.50.5

0.5

0.5

1.514.51620943165.50.5

1.514.51620943165.50.5

1.51216188.543165.50.5

2.520.5

1

1

1.520.5

1

1

0.5

0.5

128.5156.5154.5213.5164170194170224.5201217173153148130

1.5340.51.51.510.510.510.510.5

33.55145152.52.53.51.5132.5

0.50.5

0.50.5

220.5180.513.5146.571.52.51

130.511.50.579450.51

7623.51.50.5

7623.51.50.5

1.50.50.5

1

60.55.50.555.52.54.50.51

60.55.50.555.52.54.50.51

0.5

5.50.55.50.555.52.540.51

96.56.552.5211.51

3.510.51.553.52.55.561.52

3.510.51.553.52.55.561.52

0.50.50.5

3.50.50.51.553.52.55.55.51.51.5

3.50.50.51.553.52.55.55.51.51.5

3.50.50.51.553.52.55.55.51.51.5

0.511.5

0.51.51

0.51.51

0.51.51

0.51.51

0.51.51

1.50.50.50.50.50.50.5

1.50.50.50.50.50.50.5

1.50.50.50.50.50.50.5

0.5

0.5

0.50.5

21.52416.56216.513.561.51071.56466.547413119

1.513.51.5131.51.530.51.50.51

1.511.510.50.50.51.50.50.50.5

0.5

0.5

0.5

0.5

1.511.510.50.510.50.5

1.511.510.50.510.50.5

0.5

0.5

1

1

0.5

0.5

22.5111.50.50.50.5

0.520.50.5

20.50.5

0.5

0.50.50.50.51.50.50.50.5

0.50.50.50.51.50.50.50.5

0.50.5

0.50.5

0.50.5

5.540.5200.5125.50.528.525232215127.5

1.50.51.5

0.50.55545.53.532.52.51

0.50.55545.53.532.52.51

0.52.53.524.5231.51.5

0.52.51.5211.5111

0.50.54.56.510.5666.52.552

10.50.5

0.50.5

2

2

2

13.52.5120.5

13.52.5120.5

0.511.5

4.530.50.519.50.510.510.512.51084.54

430.50.5159786423.5

430.50.5159786423.5

0.5312.52.52.522.50.5

1.50.50.5121.52

1.510.5

0.5

0.5

8.5

5.5

5.5

2

1

0.5

0.5

0.5

1.5

1.5

1

4.51.50.5250.526232215.510.512.554.5

0.5

70.57.54.55.543.5410.5

0.50.50.5

0.50.50.5

6.5

0.5

0.5

0.5

0.5

7.54.55.53.533.510.5

5.5

0.5

0.5

5

5

0.50.50.50.5

465.5432

465.5432

1.53.51.5110.5

0.51.50.51

10.51

1.50.50.51.521.531.52.5111.5

1.533431.51.521.5

1.533431.51.521.5

1.5333.531.51.521.5

0.5

1166.56.54.542.53.510.5

1166.56.54.542.53.510.5

0.5

0.50.50.50.50.5

1366.543.52.5310.5

0.52.5

11.5171413.514.51179.51815.524.5141213.56

233332.50.521.533.51.53.51.52

121.50.50.5211.51.51.512.51.520.5

0.50.50.51.50.50.5

0.50.50.51.50.50.5

121.50.50.520.51.5111111.50.5

10.50.521.50.5

0.50.50.5

0.50.50.50.50.511111.50.5

0.5

2110.50.510.510.5

2.53.51.520.51547332.51.5

2.53.51.520.51547332.51.5

2.53.51.520.51547332.51.5

1.513.51.524141.521.51

0.5

0.5

1.513.51.523.5141.521.51

1.511.523.5141.521.51

3.5

10.50.50.511.5

1.53.550.552.52.510.5110.53.50.5

2.511.530.52.5213.5363.511.5

0.5

0.5

0.511.50.52.5110.5

0.511.50.52.510.50.5

22.523.525.53.511.5

0.521.50.520.5

1211.531.511.5

0.50.510.51.5

1.50.5212.51

1.50.5212.51

1.50.5212.51

0.51.520.511.520.50.5

0.51.520.511.520.50.5

0.5

0.5

0.5

0.5

0.51.520.50.5110.5

10.5

44.5422.51.50.51.521.531.52.52.54

1.534320.520.50.5120.5

0.5

283.51.57.550.58.52.51.5321.54.54.5

0.5

0.5

0.5

0.5

110.510.511

20.50.57.50.57.576.5653.54.52

0.510.50.50.5

0.510.50.50.5

0.510.50.50.5

0.510.50.50.5

0.51.511.5

0.51.511.5

0.511.5

0.51

0.51

0.51

21.50.510.50.5

21.50.510.50.5

21.50.510.50.5

0.50.5

1.5110.50.5

1.50.50.530.545545342

2119.5104397.540.57.5442836.52734.52228

1515.571686.51762611.521.511.520.51519

3.51.51.512.511189.511.568.54.55

3.51.51.512.511189.511.568.54.55

30.51.51017.56.53.564.5533

0.5122.51.55.55.51.53.51.52

1.50.50.5

0.510.521.55.55.51.53.512

0.50.5

0.50.5

1.50.5

0.51.50.5221.53133.50.53

13.53.53211.50.5

13.53.53211.50.5

13.53.53211.50.5

13.53.53211.50.5

331.50.5

331.50.5

10.5

2310.5

2310.5

1.51.510.51.5

0.5110.510.51.511.51.50.511

0.510.51.50.50.50.5

0.50.50.51.5

0.511

0.50.5

0.50.5

0.50.5

0.5

0.5

3

3

3

3

3

3.55.55.5987.59.511.59.59.51055.563.5

2.51.556.544.5811.56.597534.52

10.5

10.5

10.5

10.52.50.5233.542.511.51

1.52.55.50.534220.52.51

30.52.52.53.50.50.5

20.52.51.51.50.5

0.50.5

20.5211.50.5

110.5

10.5

0.510.50.50.50.50.50.50.5

0.510.51.57

10.51

0.50.50.5

140.52431.52.50.52.521.51.5

0.5

140.51430.50.5221.51.5

140.51430.50.5221.51.5

0.50.50.5

0.511.5

0.511.5

0.5

0.50.50.5

0.50.50.5

0.5

1010.529.51.525.54913.515.512.59125.5

1010.529.51.525.54913.515.512.59125.5

1010.529.51.525.54913.515.512.59125.5

108.581150.581314.510.589.55.5

4751120.53464.5343

61.533598.5655.52.5

221.50.50.50.53.510.51212.5

311.551.5353.53.56.575551.5

0.5

0.5

0.5

0.5

111.50.510.510.5

0.510.50.5

0.50.5

0.50.5

0.50.5

0.50.5

0.5

0.50.50.510.50.51212.531.51.52.5

0.50.50.510.50.51212.531.51.52.5

0.50.50.52

0.50.511210.511

10.5

10.5

10.5110.51

0.5

10.510.50.51

1.50.50.520.50.541.52.53.53.522.51

0.51.50.5

0.5

0.5

1.50.520.50.541.52.523.51.52.51

10.50.50.50.521.50.50.50.51.50.5

0.51.5221.531.510.5

0.51.5221.531.510.5

10.50.5

46.542.58.51016.521.51.51.523.54

46.542.58.51016.521.51.51.523.54

46.542.58.51016.521.51.51.523.54

46.541.58.5100.56.51.51.51.51.523.54

3531.5890.56.51.51.51.51.51.53.54

11.510.510.5

10.50.5

10.50.5

810.5207.520235.523.57.5478.56.579

1.50.50.5

2.5130.52.50.5

1.510.56145243.5413

1.510.56145243.5413

1.510.56145243.5413

0.520.50.51.510.50.51

0.50.5

10.50.50.5

1.50.50.52.511211.512.50.52

10.5

1

5.56.5161.516.5180.519.52.52242.565.5

0.50.50.50.50.50.5

0.5

0.5221.50.5

0.50.50.50.51

0.5

1140.553.52.51.51

0.50.5

0.5

0.5

2385.59.58.51.510.512.523

10.5221.510.50.5

1122160.50.5

0.50.5

27.5394923.55563.52562.528.5293331.52024.525.5

1111.52.53.511

2.51.51.51.5434.53.52.531.50.53

2.51.51.51.5434.53.52.531.50.53

2.51.51.51.5434.53.52.531.50.53

2.51.51.51.5434.53.52.531.50.53

0.50.523.52.540.5

14

14

14

14

6.513.52836.54624135.54.53254.5

1243.53.5

0.51

0.50.513.52.510.5

11.53.551.5210.521.511.50.5

0.5

13.59.51827.524.52221221.5

35.5127.597.50.50.50.511.5

10.52013.510.59.5127.515121819.511.51613.5

10.52013.510.59.5127.515121819.511.51613.5

10.52013.510.59.5127.515121819.511.51613.5

1019.513.510.59.5127.515121819.51115.513.5

0.5

6.542810.570.56886433

0.50.50.510.50.50.51.50.5

0.50.510.50.50.51.50.5

0.50.5

0.50.5

0.50.50.50.51.50.5

0.50.50.51.50.5

10.5

0.5

0.5

0.5112

11.50.50.51.5

0.50.513.5

0.50.513.5

0.50.513.5

0.5

0.50.513

0.50.513

651101271109.58.56.551010.5

10

10

1.5

1.5

1.5

2

5

0.50.511

0.50.511

0.50.511

5.5511270.598.58.56.551010.5

5.5511270.598.58.56.551010.5

11.51.51.50.520.5

3.520.51210.52.51.54234.57

4.521.50.51.50.51.5

220.51.5342.52.51.523

220.51.5342.52.51.523

000000000000000

000000000000000

000000000000000

000000000000000

000000000000000

000000000000000

000000000000000

000000000000000

000000000000000

000000000000000

000000000000000

000000000000000

168230.5276.534238.5252.529304.555.57896122149153.5186.5

23.557105.53.590128.52.518274.56.554.524.526.5

19.55197.583.51211171.53.5254.5217.521

0.52.540.53.54.51.56.51.51.5122.5

2.53.520.510.532.5

2

111.510.50.511.5

111.510.50.511.5

111.510.50.511.5

111.510.50.511.5

143.517217030.5147.5122.526.5121.548.57389115144.5127.5159

465873955517.554.51318.522.525.556.538.562.5

3747.563.574845.55.55281416.52046.536.553.5

0.50.5

0.50.5

3747.563.56.54845.55.551.5813.516.52046.536.553.5

0.512.52.510.50.50.50.50.50.50.5

36.546.5616.545.544.55.5517.5131620463653

1.5

1.5

1.5

0.50.51.5

0.50.51.5

0.50.51.5

8.510.55.554213.54.565.51029

8105.554213.54.565.51029

8105.554213.54.565.51029

0.50.5

3142.536.512.5282614.520.51625.51529.5252428.5

3142.536.512.5282614.520.51625.51529.5252428.5

0.5

110.51.50.50.5

33215.54.520.50.5211.524

10.511.5

2837.53211.521.520.514.51715.523.512.527.5232024.5

1724.520315166.5136.5105.51113711

65.571.560.5964.545.54.54619.52951606364.568

0.526770.5

65.57158.5958.538.54.53919.52951606364.567.5

19.51.53.5212.52.5

57.5555225225.51.521.51519384855.55762

0.510.51.510.5

153.513101410.511

11131.50.5111.521.530.50.5
