# Supplementary figures and images for: Shifts among Eukaryota, Bacteria, and Archaea define the vertical organization of a lake sediment
Source: Microbiome. 2017 Apr 8;5:41. doi: 10.1186/s40168-017-0255-9 (PMC5385010; doi:10.1186/s40168-017-0255-9)

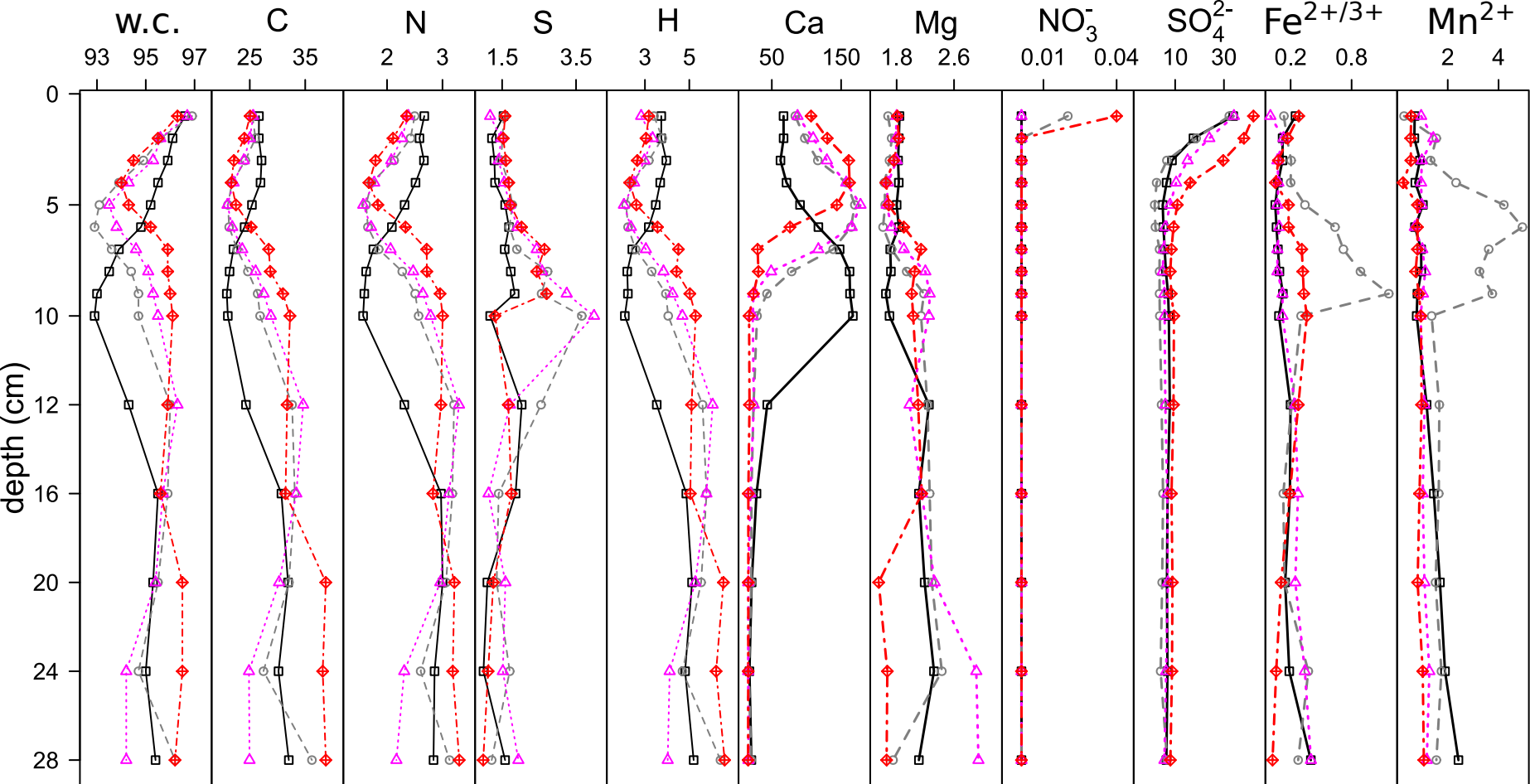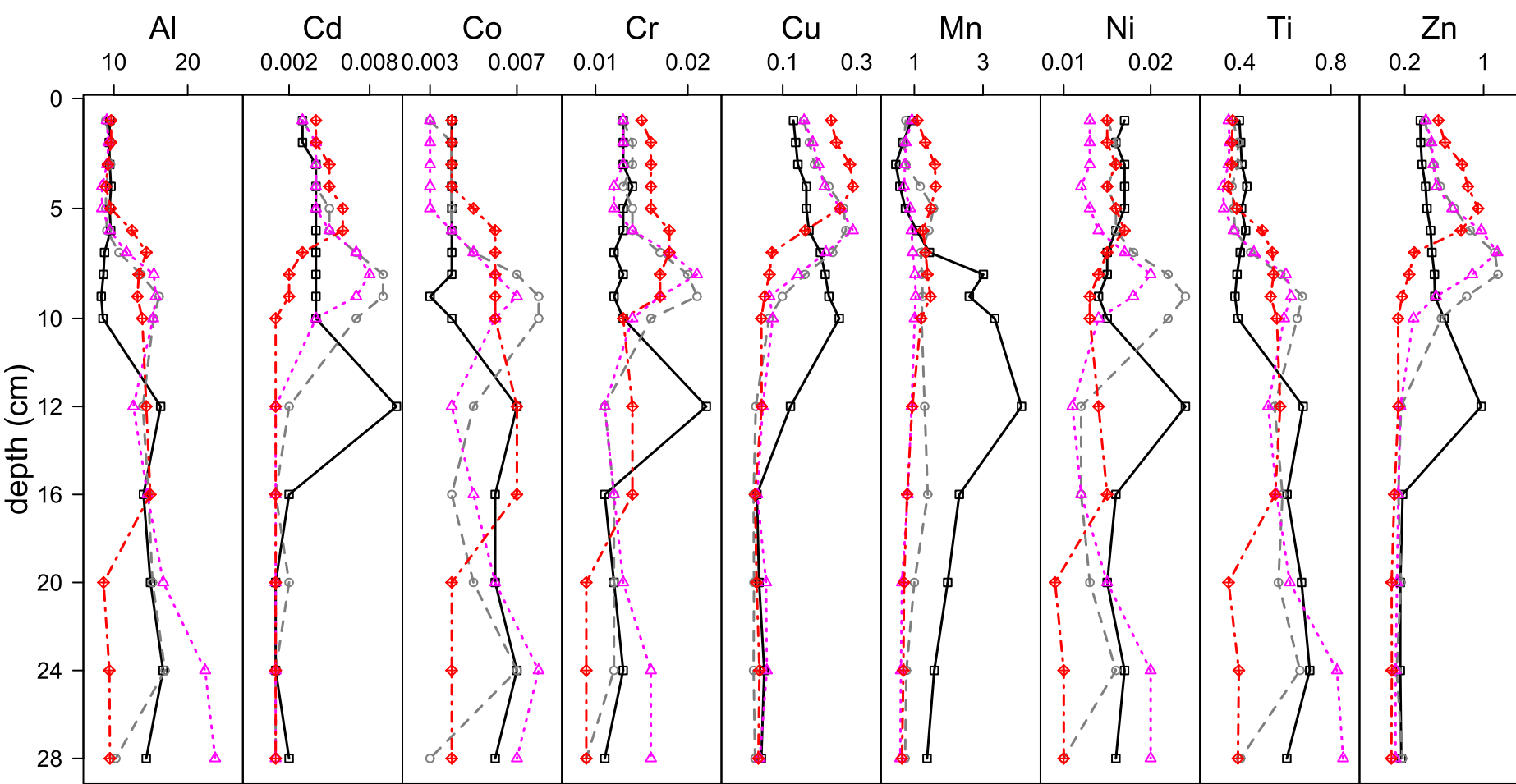

—□— A —○— B —△— C —◇— D

Supplement: Supplementary file 2 — Figure: detailed depth profiles of individual cores. Additional detailed depth profiles of individual core (A,B,C,D) variables of Lake Stechlin at 30-m depth. Units: w.c.(water content) [%]; C [%]; N [%]; S [%];H [%]; Ca [mg g −1 dry weight]; Mg [mg g −1 dry weight]; NO$_{3}^{-}$3− [mg l −1]; SO$_{4}^{2-}$42− [mg l −1]; Fe 2+/3+ [mg l −1]; Mn 2+ [mg l −1]; Al [mg g −1 dry weight]; Cd [mg g −1 dry weight]; Co [mg g −1 dry weight]; Cr [mg g −1 dry weight]; Cu [mg g −1 dry weight]; Mn [mg g −1 dry weight]; Ni [mg g −1 dry weight]; Ti [mg g −1 dry weight]; Zn [mg g −1 dry weight]. See [108] for comparison with previous data. (PDF 91 kb) [file 40168_2017_255_MOESM2_ESM.pdf]

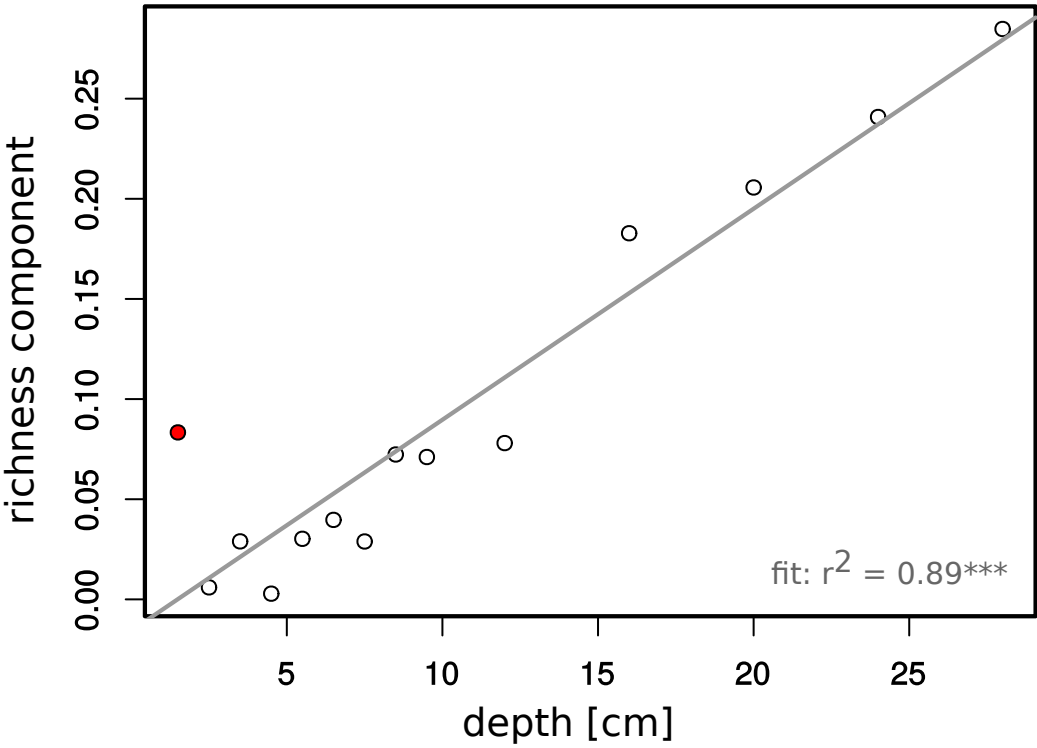

Supplement: Supplementary file 3 — Figure: richness component vs. depth. Increasing richness component with increasing depth. The first cm is an outlier of the observed linearity. (PDF 15 kb) [file 40168_2017_255_MOESM3_ESM.pdf]

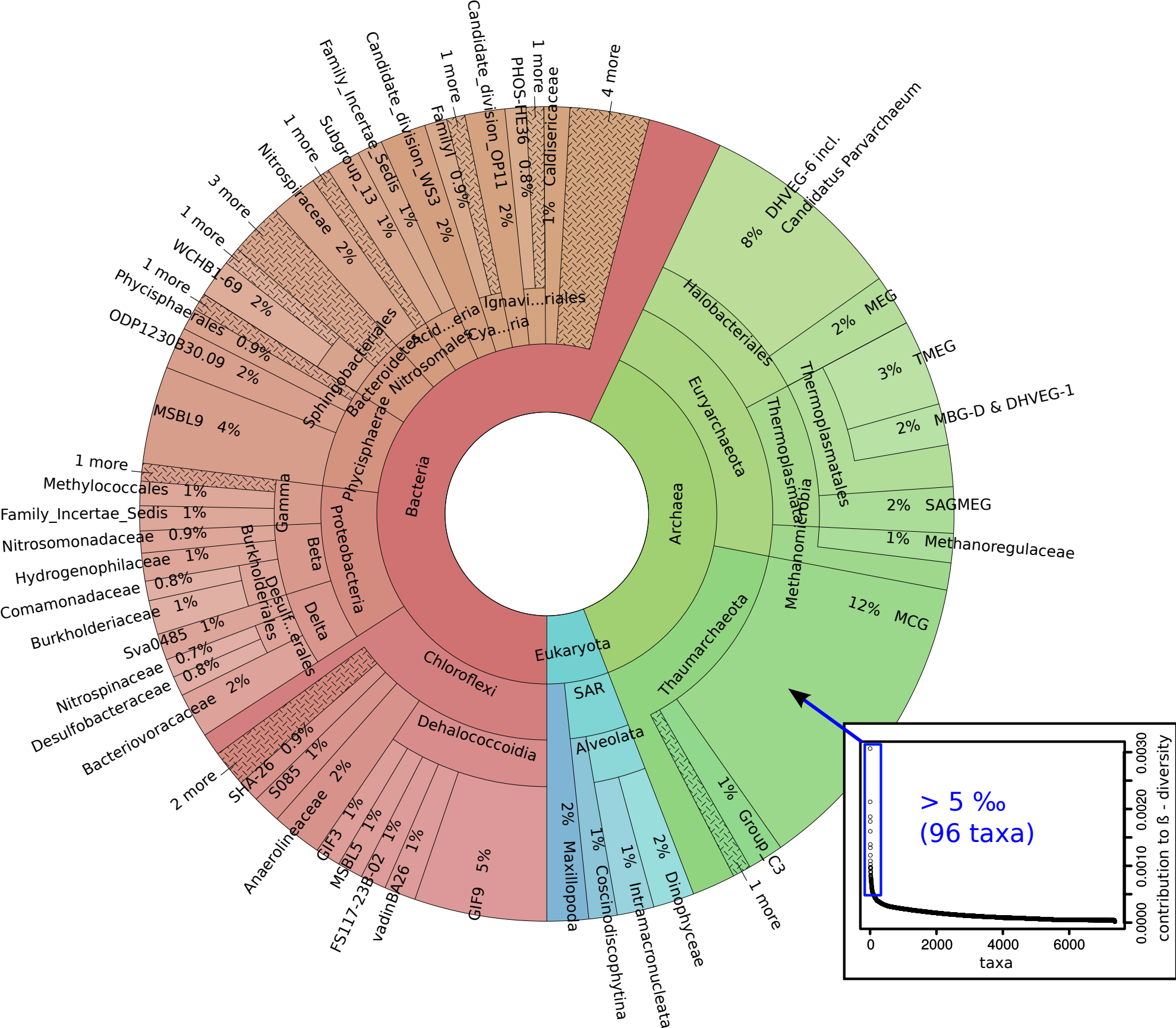

Supplement: Supplementary file 4 — Figure: taxonomic composition of the most structuring taxa. Hierarchical taxonomic presentation of the most structuring taxa (SCBD), i.e., all OTUs that account for more than 5 per mill of the total β-diversity (see inlet to the left). The pie chart is color coded according to the three domains: Bacteria (red), Archaea (green), and Eukaryota (blue). (PDF 197 kb) [file 40168_2017_255_MOESM4_ESM.pdf]

observed DNA values

0 20 60 100

0

20

40

60

80

100

predicted DNA concentrations

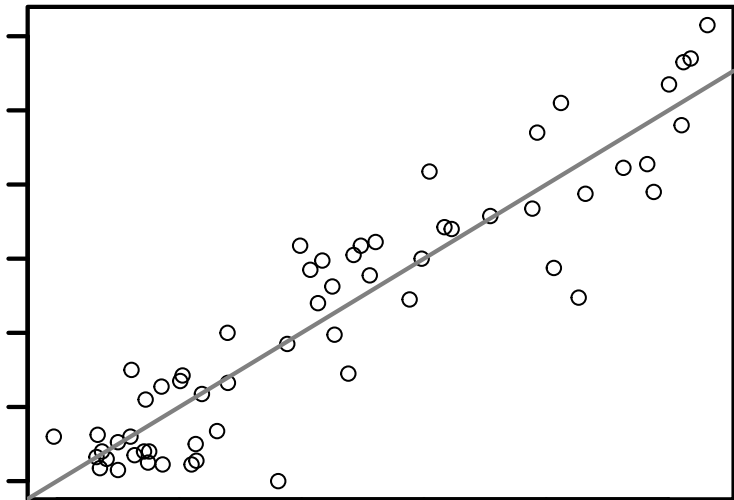

Supplement: Supplementary file 5 — Figure: sediment DNA as a function of present taxonomic signals. Multiple linear regression on the sediment DNA content as a function of the occurrence of Eukaryota (75.6% of the variation) together with Bacteria (10.0% of the variation; model: R 2=0.856, p<0.001). (PDF 10 kb) [file 40168_2017_255_MOESM5_ESM.pdf]

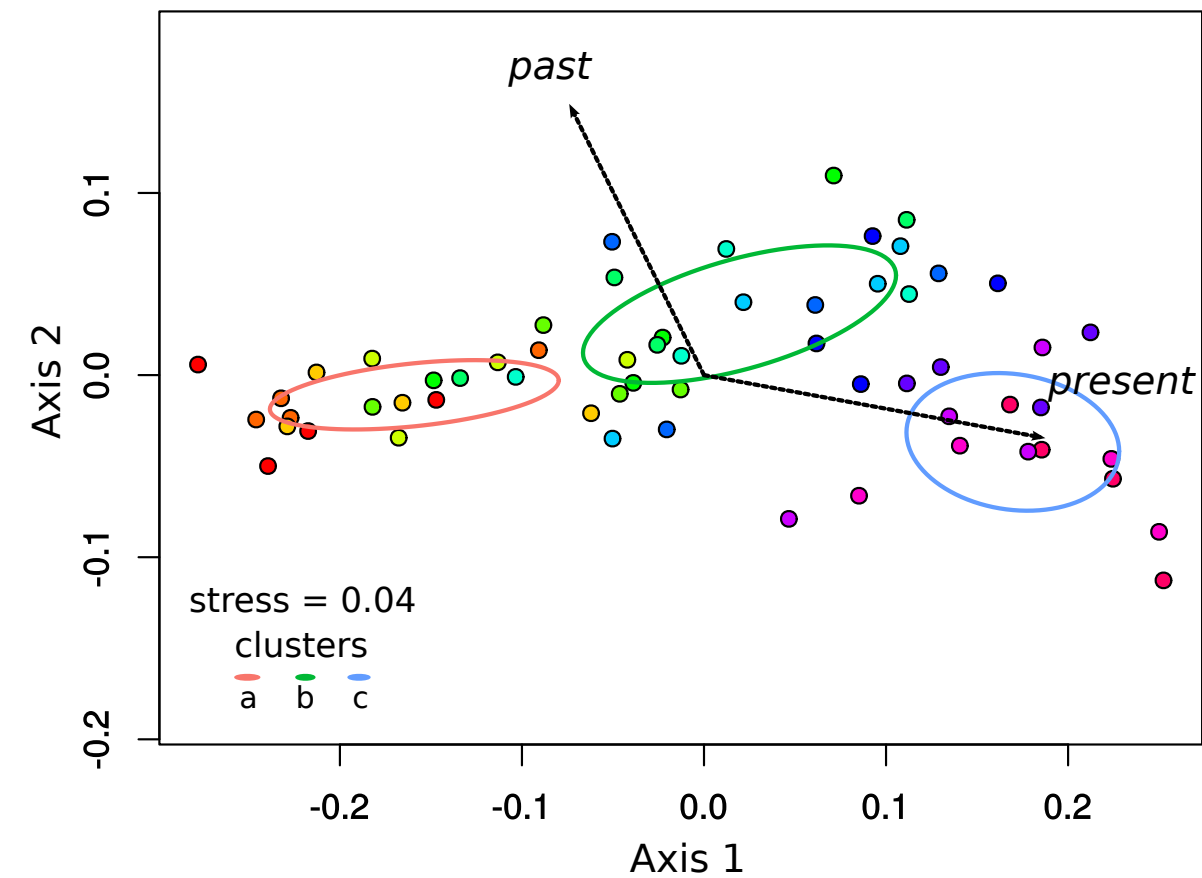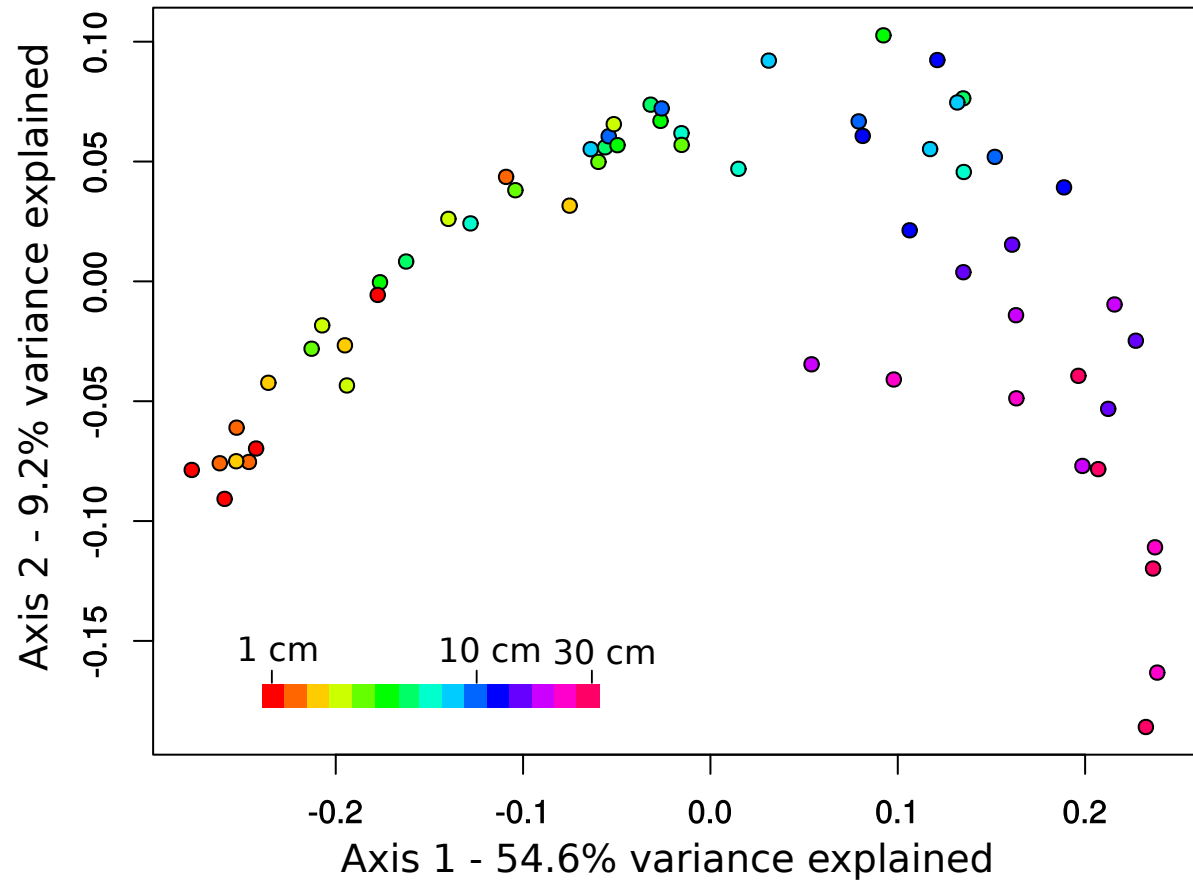

Supplement: Supplementary file 6 — Figure: UniFrac ordinations. Left panel - A nonmetric multidemsional scaling (analogous to Fig. 4 b) of all the samples based weighted UniFrac distances. This was also reflected in the distance between the surface and deep sediments on axis 1 (adonis: R 2=0.520,p<0.001). We were able to significantly recover the three depth zones (adonis: R 2=0.601,p<0.001). The overall community structure was correlated with both present (Mantel correlation: r=0.512,p<0.001) and past (r=0.333,p<0.001) parameters, which were nearly orthogonal in ordination. Right panel—a metric multidimensional scaling (principal coordinate analysis) of the UniFrac distance matrix that is displayed in Fig. 3 b, with the corresponding proportional eigenvalues for each axis. The curved shape may point to an ordination artifact. (PDF 27 kb) [file 40168_2017_255_MOESM6_ESM.pdf]

Depth [m]

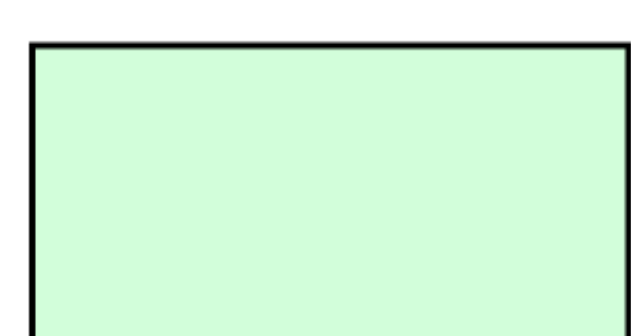

5 - 10

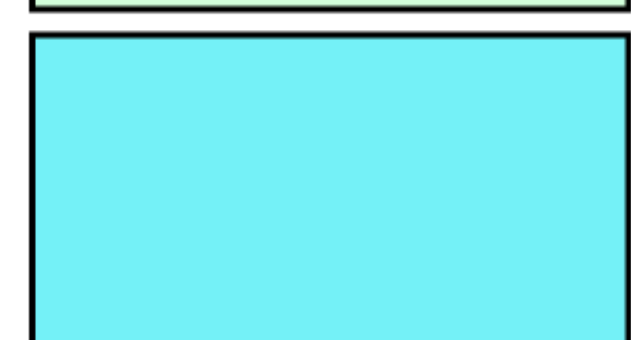

10 - 25

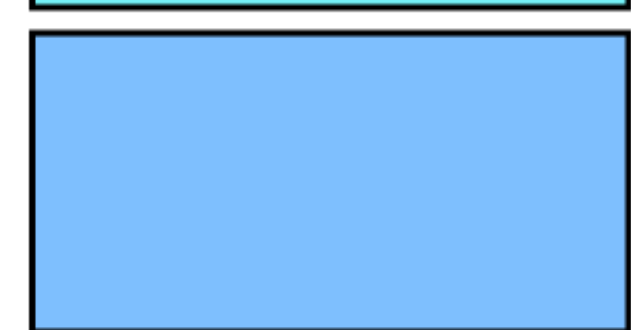

25 - 40

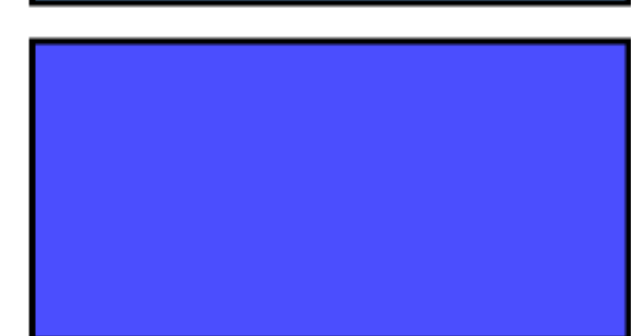

40 - 55

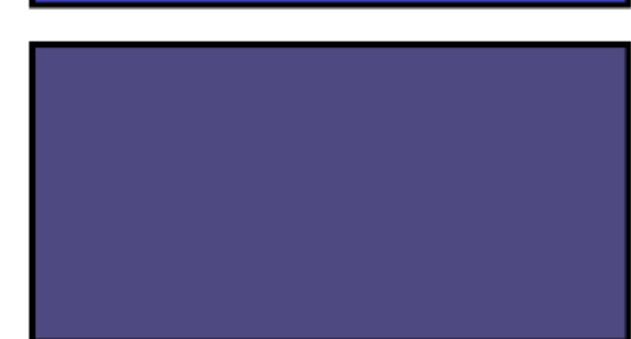

55 - 68

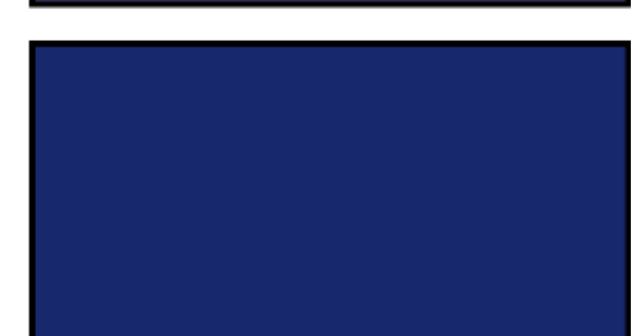

69

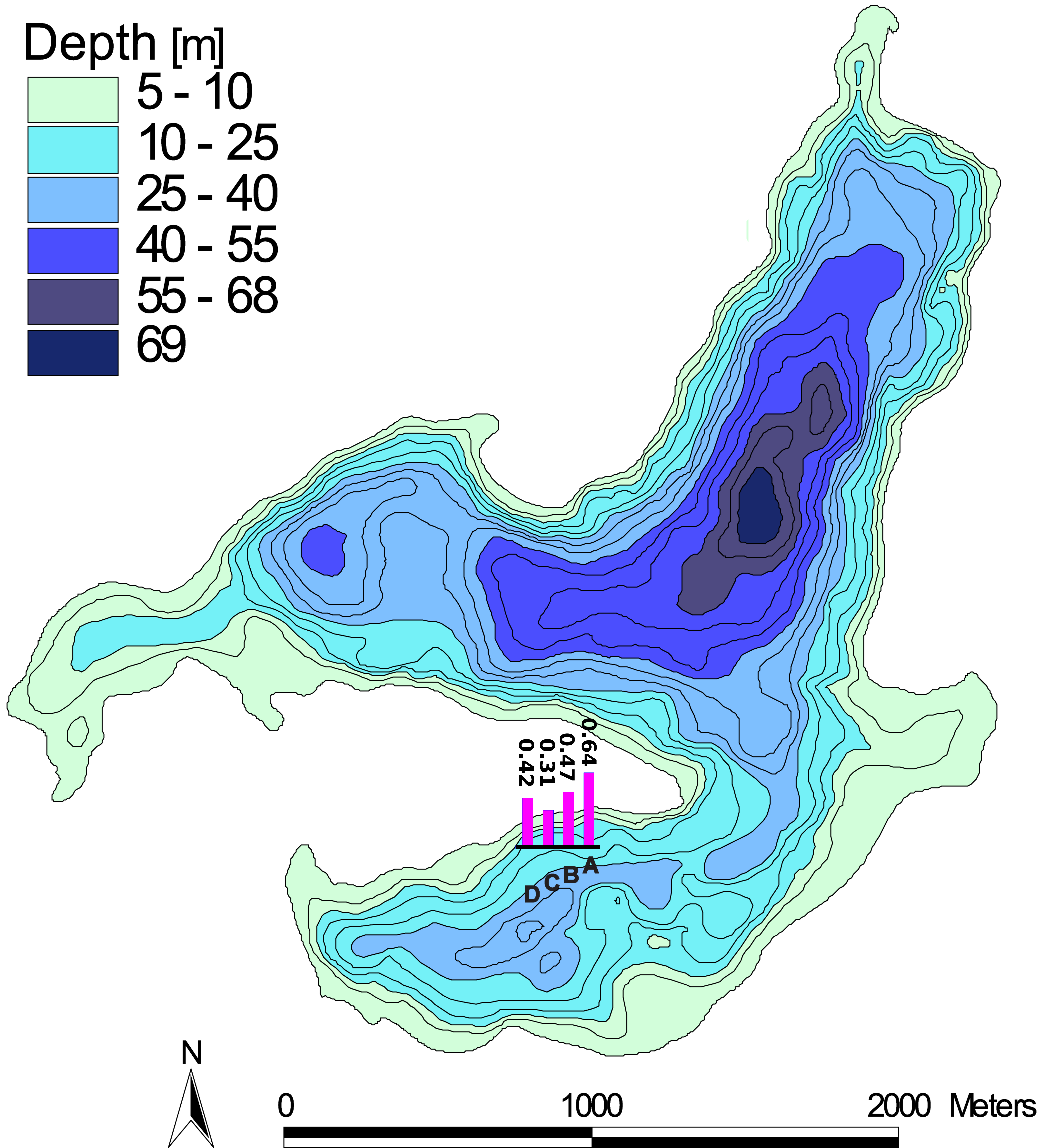

Supplement: Supplementary file 8 — Figure: DNA size distribution after extraction. DNA microgel electrophoresis (Experion, BioRad) from a random subset of samples from various sediment depths, showing the absence of small environmental DNA (<1 kb). (PDF 395 kb) [file 40168_2017_255_MOESM8_ESM.pdf]

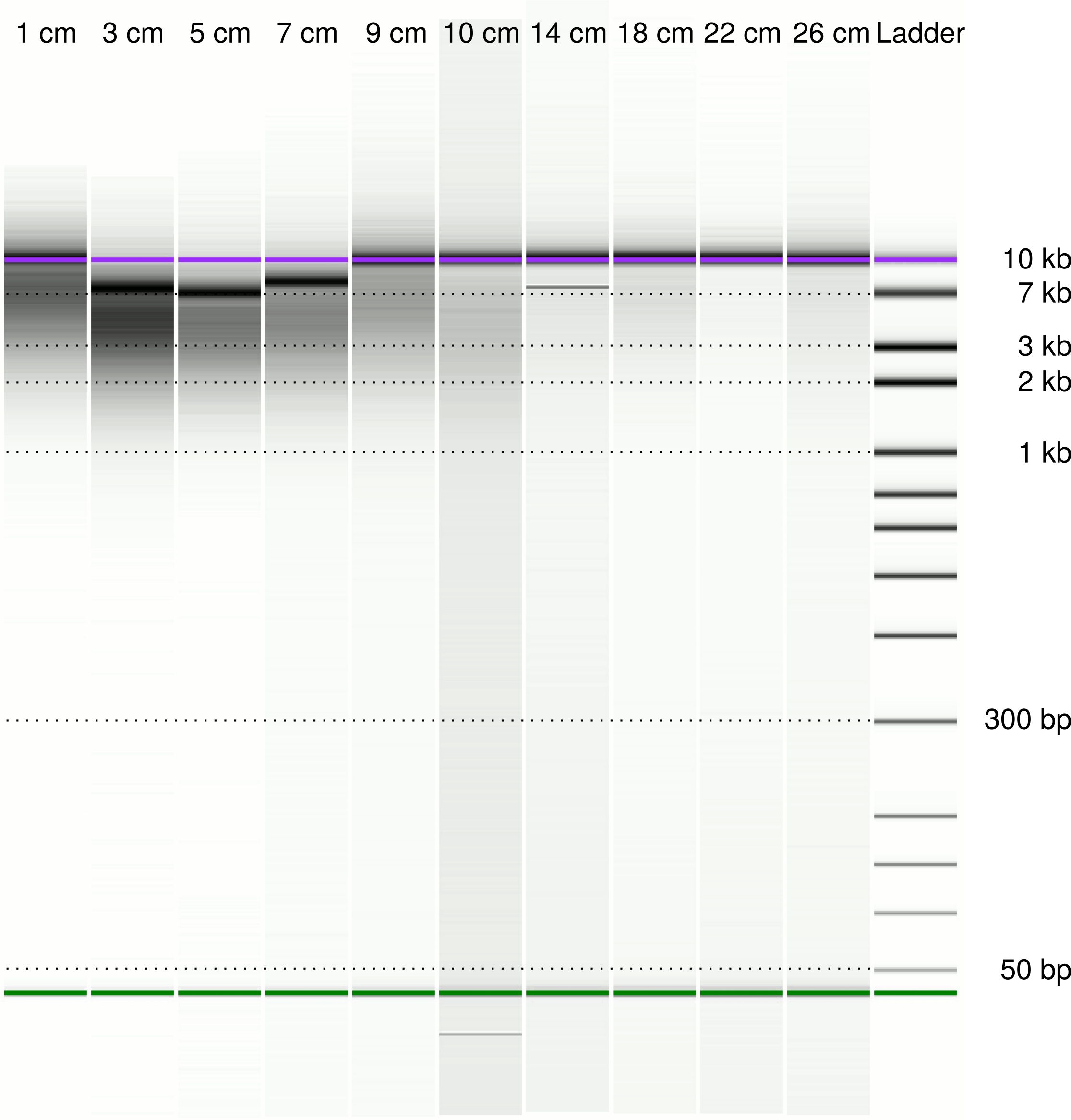

Supplement: Supplementary file 9 — Figure: sampling locations within Lake Stechlin, Germany. Depth map of Lake Stechlin (Germany) and the four replicate sampling sites (A,B,C,D) in the South-West bay with the corresponding oxygen penetration depth in cm (pink bars). (PDF 389 kb) [file 40168_2017_255_MOESM9_ESM.pdf]

present factors

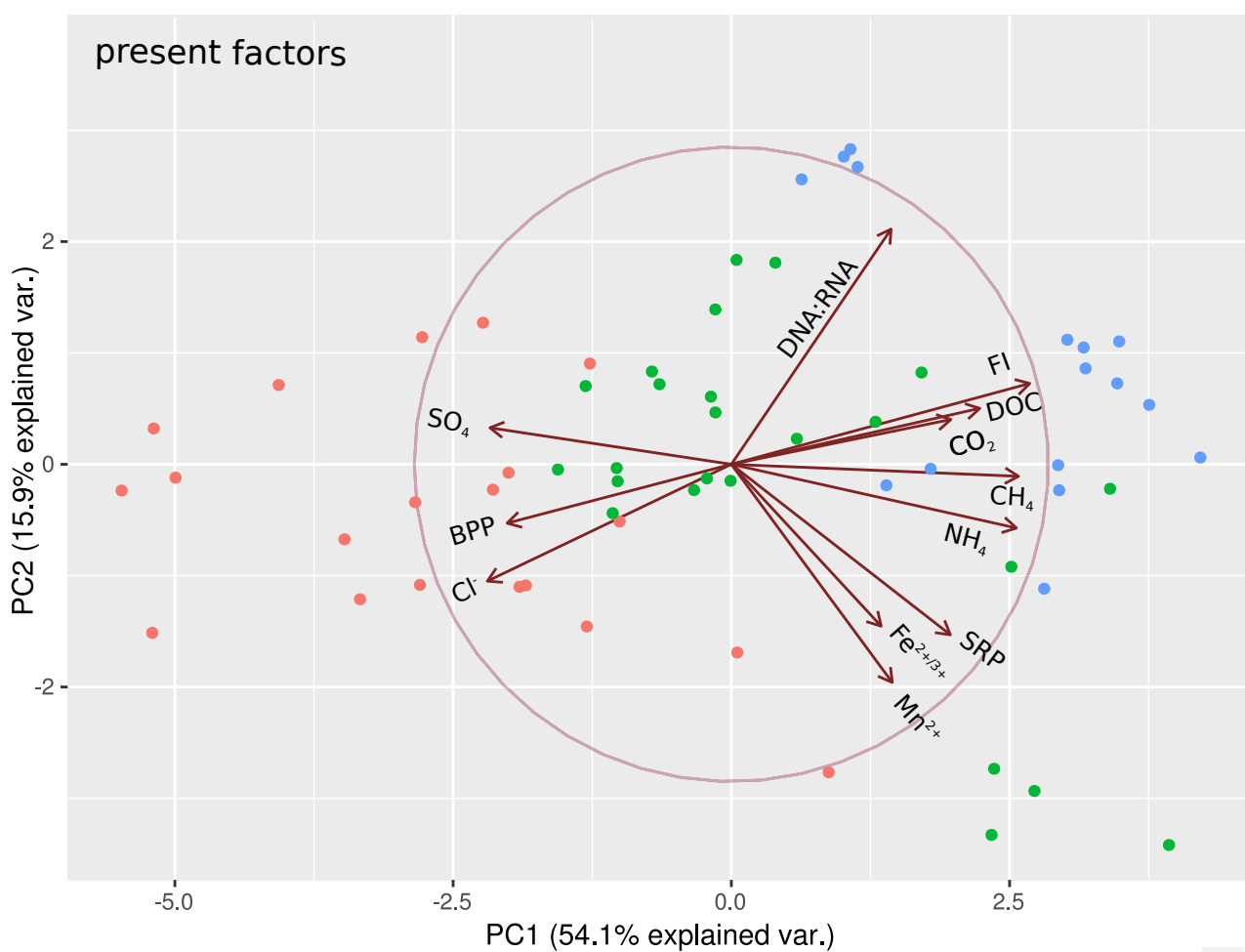

past factors

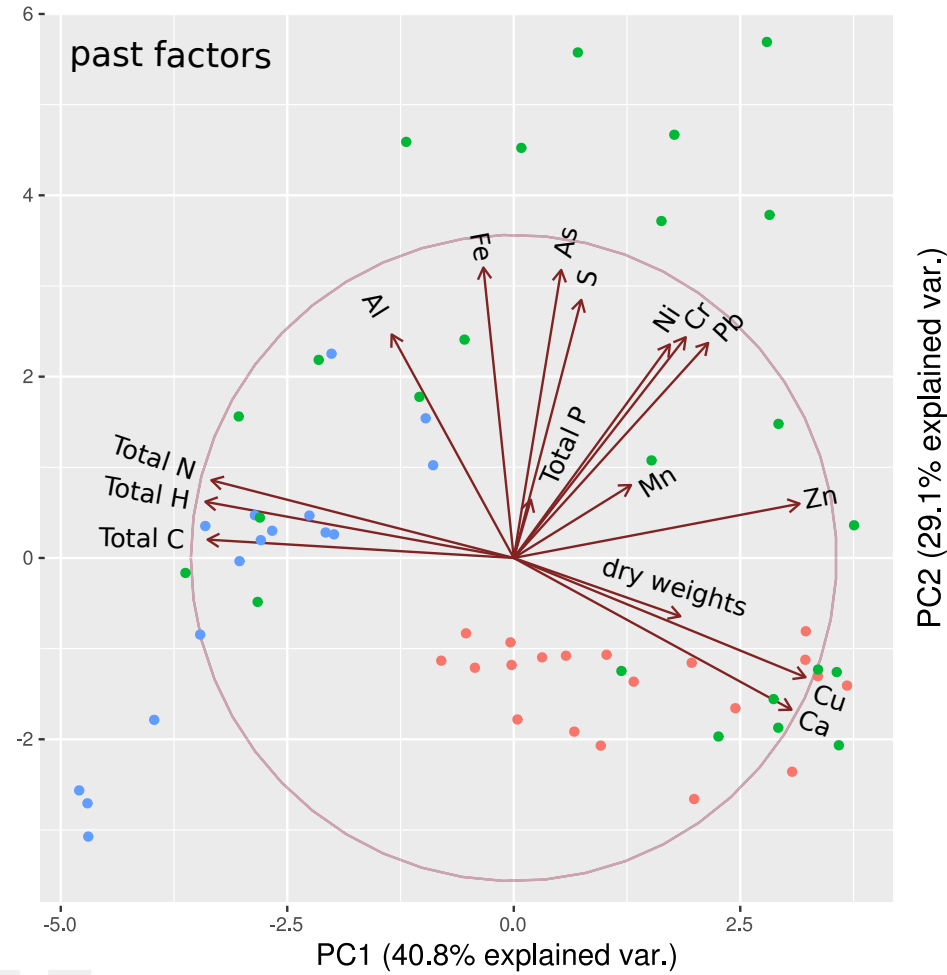

Supplement: Supplementary file 10 — Figure: principal component analysis of environmental parameters. Principal component analysis defining the “present” (left panel) and “past” (right panel) parameters. The samples are color coded according to the three depth clusters (a–c). (PDF 52 kb) [file 40168_2017_255_MOESM10_ESM.pdf]
