# Supplementary material for: Shifts among Eukaryota, Bacteria, and Archaea define the vertical organization of a lake sediment
Source: Microbiome. 2017 Apr 8;5:41. doi: 10.1186/s40168-017-0255-9 (PMC5385010; doi:10.1186/s40168-017-0255-9)
Supplement: Supplementary file 7 — Text: sediment zonation according to taxonomic clustering, β-partitioning, and context data. (PDF 14 kb) [file 40168_2017_255_MOESM7_ESM.pdf]

Information Box Our results pointed to three depth clusters. Based on the high replacment (Table 1) and measurable microbial production in the first two depth clusters (cluster a, b) (Figure 1), we decided to classify them into one overarching horizon, which we called the replacement horizon. Correspondingly, we classified the lowest depth cluster (cluster c) into the depauperate horizon, based on the loss in richness measures and constancy in most sediment parameters

- I. The replacement horizon ($1-14\ cm|ca.\ 0-60\ a$) is subdivided into two zones, delineated by the sulfate-methane transition.
  - Cluster a) redox-stratified zone ($0-5\ cm|ca.\ 0-20\ a$). This zone encompasses the redox-stratified zone and the typical sequence of electron acceptors from oxygen to sulfate. NH_4^+ and SRP increases. It is characterized by high microbial activity, cell numbers, taxa turnover, spatial variability, low DNA:RNA ratios, and low FI values ($1.7-1.8$). Potentially 50% of the sedimented organic matter is metabolized in this horizon, aligning with the rapid decrease of eukaryotes. Bacteria dominate this horizon.
  - Cluster b) transition zone ($5-14\ cm|ca.\ 20-60\ a$). This zone is characterized by strong gradients. Microbial cell numbers drop off and activity decreases, diversity decreases, the taxa turnover stays high, the DNA:RNA ratio doubles, and FI values increase ($1.8-1.9$). Methane concentrations rise, and CO_2 has a local minimum. Potentially, another 35% of the sediment organic matter is turned over in this horizon. Eukaryotes approach zero, Bacteria decline, and Archaea rise rapidly in their community contribution. Here, we also see a maximum of taxa with no close relatives to known database entries.
- II. The depauperate horizon ($14-x\ cm|ca. > 60\ a$) is very distinct from horizon I, and it is unclear how far this horizon reaches. It appears to be Archaea-dominated and is characterized by a loss in richness and a shift toward the dominance of single taxa.
  - Cluster c) ($14-30\ cm|ca.\ 60-150\ a$). The zone is characterized by constancy in most parameters, while we see significant taxon richness effects and marked drops in spatial variability, evenness, and diversity. Cell numbers and activity stay consistently low, while DNA:RNA ratios and FI values (2.0) stay consistently high. CH_4 and CO_2 approach saturation. Archaea are dominant.
